# Optimizing ultra-rapid compressed-sensing MPRAGE acquisitions for brain morphometry

**DOI:** 10.3389/fnimg.2025.1718444

**Published:** 2026-01-02

**Authors:** Lindsay C. Hanford, Tom Hilbert, Tobias Kober, Randy L. Buckner, Ross W. Mair

**Affiliations:** 1 Center for Brain Science, Harvard University, Cambridge, MA, United States; 2 Athinoula A. Martinos Center for Biomedical Imaging, Department of Radiology, Harvard Medical School, Massachusetts General Hospital, Charlestown, MA, United States; 3 Department of Psychology, Harvard University, Cambridge, MA, United States; 4 Swiss Innovation Hub, Siemens Healthineers International AG, Lausanne, Switzerland; 5 Department of Radiology, Lausanne University Hospital, University of Lausanne, Lausanne, Switzerland; 6 LTS5, École Polytechnique Fédérale de Lausanne, Lausanne, Switzerland

**Keywords:** magnetization prepared rapid acquisition gradient echo, T1 weighted, rapid MRI methods, compressed sensing, brain morphometry

## Abstract

**Purpose:**

Compressed-sensing (CS) methods can decrease the acquisition time for T_1_-weighted (T_1_w) structural MRI images to 1–2 min. Rapid acquisitions reduce participant burden, reduce the risk of motion artifacts, and allow for repeat scans to be acquired within a session. This study investigated the tradeoffs of sparse sampling and CS image reconstruction for brain morphometric applications.

**Methods:**

Magnetization-Prepared Rapid Gradient Echo (MPRAGE) images were acquired at 1.0 mm spatial resolution. The effects of the acceleration factor (x2 to x8) and regularization factor were examined. Subcortical volumes and regional cortical thickness estimates of brain structure were obtained for all T_1_w images. Within-sequence agreement was evaluated by comparing estimates obtained using the same protocol in the same imaging session. Between-sequence agreement was evaluated by comparing estimates from a fully sampled MPRAGE protocol to the novel CS-accelerated MPRAGE protocols within the same session.

**Results:**

Higher acceleration lowered the SNR in white matter but not in gray matter. SNR could be further manipulated by the regularization parameter. Within-sequence agreement was comparable across all protocols. In fact, the spread in estimates from the 58-s CSx8 protocol was similar to those from the fully sampled protocol. Similarly, high agreement was found between estimates from the fully sampled and under-sampled protocols for all acceleration levels up to eight. Modifying the regularization factor had a quantifiable effect on image smoothness, however it had minimal impact on the agreement of morphometric estimates.

**Conclusion:**

Accelerated CS imaging protocols show comparable performance to traditional longer protocols for morphometric brain estimates.

## Introduction

Morphometric analysis of brain structure is a central point of interest in the study of development (Casey et al., 2018; Harms et al., 2018; Somerville et al., 2018), aging (Jack et al., 2008; Li et al., 2021; Bookheimer et al., 2019; Miller et al., 2016; Nobis et al., 2019; Elliott et al., 2023), neuropsychiatric illness (Demro et al., 2021; Thompson et al., 2020), and for investigating individual differences (Harms et al., 2018; Lerch et al., 2017; Evans et al., 1994; Glasser and Van Essen, 2011). Nearly all functional MRI studies acquire a structural MRI image for volumetric and surface-based anatomical registration, and many utilize the anatomical details for reference during analysis. Currently, a structural T_1_-weighted (T_1_w) image takes approximately 4–8 min to acquire with conventional cartesian sampling methods, however novel accelerated-imaging techniques can reduce acquisition time to as little as 1–2 min. Such time-savings can reduce participant burden as well as reduce the risk of motion artifacts. It also becomes feasible to adopt cluster-scanning methods - where many scans are acquired in rapid succession - improving the precision of morphometric estimates (Nielsen et al., 2019).

Historically, T_1_w imaging protocols employed SPoiled Gradient Recalled Echo [SPGR (Frahm et al., 1986); also known as Fast Low Angle SHot or FLASH (Crawley et al., 1988)] techniques and took up to 10 min to acquire an image with 1 mm isotropic resolution (Jack et al., 2008; Kruggel et al., 2010; Weiner et al., 2017). Since then, the use of Magnetization-Prepared Rapid Gradient Echo (MPRAGE) (Mugler and Brookeman, 1990; Runge et al., 1991) techniques have been more widely adopted. This method adds an inversion pulse to the rapid low-flip-angle readout train of the SPGR, further improving contrast between the gray and white matter neuroanatomical features, but with no impact on scan length. Innovations to the MPRAGE technique have included: a wider acquisition bandwidth to reduce susceptibility distortion with multiple echoes (MEMPRAGE) (van der Kouwe et al., 2008), and additional readout trains following inversion to produce multiple images with varying contrast levels to remove the B1 bias field (MP2RAGE) (Marques et al., 2010). While these sequence variations further improve image contrast properties, they rather maintain or increase acquisition length, an undesirable side-effect sometimes mitigated by the use of non-isotropic voxels (Sheline et al., 1996).

Reductions in scan length became possible through the upgrade to parallel-array head coils (Wiggins et al., 2006), leading to parallel imaging through techniques like SENSitivity Encoding (SENSE) (Pruessmann et al., 1999) and Generalized Autocalibrating Partially Parallel Acquisitions (GRAPPA) (Griswold et al., 2002). As an example, two-fold in-plane acceleration (GRAPPA x2) reduces the scan length from 9–10 min to 5–6 min for an image with 1 mm isotropic resolution. T_1_w structural imaging with GRAPPA acceleration has become widely adopted [including by the Alzheimer’s Disease Neuroimaging Initiative (ADNI) (Jack et al., 2015; Jack et al., 2010)] and is generally recommended for morphometry (https://surfer.nmr.mgh.harvard.edu/fswiki). However, in-plane acceleration beyond a factor of two or three creates images that suffer from poor Signal-to-Noise Ratio (SNR) and aliasing artifacts. Additionally, increasing in-plane acceleration (from GRAPPA x2 to x4 or more) requires an increase in the amount of central-k-space reference lines acquired, so the time-savings are limited (Hoge and Polimeni, 2016; Weller et al., 2013).

Accelerated imaging protocols using Compressed-Sensing (CS) methods allow for the acquisition of a T_1_w image with 1 mm isotropic resolution in as little as 1–2 min. The two main CS-related imaging parameters available for user manipulation include the acceleration (or under-sampling factor), and the regularization factor, which is important for image reconstruction. The CS method employed here, developed to accelerate MP2RAGE scans (Mussard et al., 2020), acquires the data through sparse, incoherent under-sampling (Lustig et al., 2007; Lustig et al., 2008). In doing so, this method minimizes the structure found in aliasing artifacts, as compared to the type of coherent artifacts produced with parallel imaging. Next, the CS image reconstruction employs a well-tuned non-linear wavelet transform algorithm to reconstruct T_1_w images from the incoherently under-sampled k-space matrix (Lustig et al., 2007; Lustig et al., 2008). Image reconstruction is an iterative process done directly on the scanner console, with the CS image reconstruction optimized through choice of the regularization parameter (Mussard et al., 2020; Lustig et al., 2007; Lustig et al., 2008). In practice, the regularization parameter influences image SNR and smoothness (Mussard et al., 2020; Lustig et al., 2007).

Despite the time since the original papers on the application of CS to MRI (Lustig et al., 2007; Lustig et al., 2008), adoption by the scanner vendors has slowed uptake of this powerful method beyond MR-physics researchers. Some recent studies have considered the benefits and drawbacks of CS acceleration in clinical applications (Jaspan et al., 2015; Sartoretti et al., 2018; Vranic et al., 2019; Meister et al., 2023; Picchi et al., 2024). Others have focused on neuroimaging applications (Mussard et al., 2020; Duan et al., 2020; Yarach et al., 2021; Trotier et al., 2022; Beaumont et al., 2023; Hübner et al., 2024; Rowley et al., 2024), some of those specifically being applied to brain morphometery (Mussard et al., 2020; Yarach et al., 2021; Hübner et al., 2024), while others have focused on T_1_-mapping approaches (Beaumont et al., 2023; Rowley et al., 2024). The HBCD study in the USA recently became the first large multi-site neuroimaging study to only acquire CS-accelerated T_1_w and T_2_w images (Dean et al., 2024), while the ADNI consortium is now exploring CS acceleration techniques on certain scanners (Arani et al., 2024). However, optimizing the tradeoffs between the under-sampling and regularization in relation to basic image quality metrics or the resulting morphometric outputs has not yet been systematically explored.

This study continued our long-running exploration of accelerated T_1_w image acquisition methods for brain morphometry (Elliott et al., 2023; Nielsen et al., 2019; Mair et al., 2013; Holmes et al., 2015; Mair et al., 2016; Hanford et al., 2021; Mair et al., 2018; Elliott et al., 2024). Recently (Elliott et al., 2023), we demonstrated the use of a CS MPRAGE protocol with x6 acceleration and default regularization in a group of older adults with and without neurodegeneration and showed comparable morphometric estimates to those obtained from conventional scans. Most recently we found that the acquisition of multiple rapid T_1_w images could significantly reduce measurement uncertainty in morphometric estimates in older adults compared to a similar amount of time being spent to acquire a conventional scan (Elliott et al., 2024). These results demonstrated the viability of highly accelerated CS sequences, while noting that default acceleration and regularization settings were likely not always optimal. To provide further insight, here we study and report on the tradeoffs between under-sampling and regularization during image reconstruction on resulting T_1_w image quality and structural morphometry, using a Research Application Sequence developed by the vendor (Mussard et al., 2020), and which is available to other research sites. First, for the implemented k-space trajectory (Mussard et al., 2020), we investigated the effects of the under-sampling (or acceleration) factor while holding the regularization factor constant. Next, each acquired image (identical k-space matrix) was repetitively reconstructed varying the regularization parameter. Image quality, within-sequence agreement, and between-sequence agreement of structural brain estimates acquired using traditional fully sampled MPRAGE images to those obtained from accelerated CS images were explored. Results revealed that highly accelerated CS imaging protocols can yield images with quantitative quality metrics and morphometric estimates that in many cases matched those of traditional imaging protocols.

## Methods

### Overview

To test the current limits of CS under-sampling in our method for T_1_w structural MRI, we acquired a set of T_1_w images starting at CSx2, increasing acceleration by 2 up to CSx10. At CSx10, excessive ringing artifacts appeared consistently. We limited further testing up to CSx8. Next, participants were recruited and completed two scanning sessions within 7 days. A total of 18 T_1_w images were acquired within each session. These included: 2 images using a fully sampled MPRAGE protocol, and 4 sets of 4 images acquired using under-sampled, CS-accelerated protocols. During image acquisition, the under-sampling factor was varied, while the default regularization setting was used for image reconstruction. Acquired CS images (identical k-space matrix) were then repeatedly reconstructed, varying the regularization parameter to test its effect on image quality and resulting brain morphometry.

### Participants

Participants were three healthy adult females between 26 and 29 years of age and with no MRI contraindications. One participant completed a single imaging session where the limits of under-sampling were initially tested. Two participants completed 2 imaging sessions within a 7-day period. Written informed consent was obtained as reviewed by the Massachusetts General Hospital Partners Human Research Committee. Participants were compensated for their time.

### Under-sampling method and image reconstruction

CS is a long-researched acceleration technique in MRI, originating from information and approximation theory (Donoho, 2006; Candes and Wakin, 2008). Despite being introduced in MRI by Lustig in 2007 (Lustig et al., 2007; Lustig et al., 2008), standardized methods available to the wider community have been slow to be developed. The accelerated MPRAGE sequence employed here is a Research Application sequence developed by the authors who are Siemens Healthineers employees. It is described fully in Mussard et al. (2020), and only a brief summary is included here.

**Figure 1:**
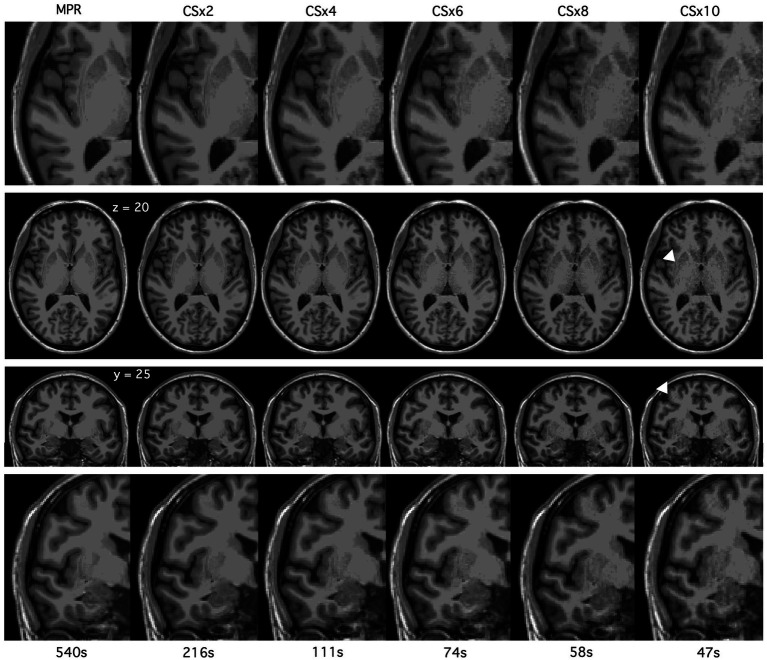
T_1_-weighted (T_1_w) images acquired using fully sampled MPRAGE (MPR) and compressed-sensing (CS) with under-sampling factor x2, x4, x6, x8 and x10 MPRAGE protocols in a pilot participant. Axial (z = 20) and coronal (y = 25) slices as well as zoomed in portions of each are shown here. Images appear more granular or noisy as lower signal and amplified noise reduce Signal-to-Noise Ratio (SNR) non-uniformly. White arrows denote ringing artifacts that were seen at CSx10 in this pilot participant and were sometimes present at CSx8.

Instead of a coherent under-sampling pattern (like it is used for parallel imaging), incoherent under-sampling is achieved using a jittered spiral phyllotaxis sampling trajectory (Mussard et al., 2020). This method calculates, first, a sampling mask using a spiral phyllotaxis pattern with a variable density. Second, k-space trajectories are derived from that sampling mask. These trajectories are rapidly computed based on a set density factor, a random jitter to promote incoherence, and standard imaging parameters such as matrix size and turbo factor. The trajectory traverses the *k_y_* and *k_z_* domain in an arcwise pattern with jitter, the randomness inherently resulting in some small variation in the sampling pattern from scan to scan. However, this samples the center of k-space in the center of the trajectory time-wise, notably allowing a user-defined number of phase-encoding steps per readout block (turbo factor), providing additional flexibility for acceleration, rather than the steps per block being equal to the number of slices acquired in the 3^rd^ dimension as in conventional scans. Example trajectories are shown in Figure 1 of reference (Mussard et al., 2020), while the reconstruction is described by Eqn. 3 in reference (Mussard et al., 2020). Reconstruction begins with a conventional Fourier Transform in the readout domain to break the 3D reconstruction into multiple 2D reconstructions for each slice in the image. A cost function relying on the sampling mask, complex coil sensitivities, and the wavelet transform scaled by a regularization factor is minimized iteratively using the scanner’s standard reconstruction computer. The regularization factor trades off image SNR and image smoothness/blurring.

### Scan acquisition

T_1_w images were acquired at the Harvard Center for Brain Science using a 3 T MRI scanner (MAGNETOM Prisma^fit^, Siemens Healthineers, Forchheim, Germany) and the vendor’s standard 32-channel head coil. For the two participants who completed repeat sessions, a total of 18 T_1_w images were collected within each session. Of those, 2 T_1_w images were acquired using a standard MPRAGE protocol. Additionally, 4 sets of 4 T_1_w images were acquired using CS protocols varying the under-sampling factor (CSx2, CSx4, CSx6, CSx8).

The two conventional MPRAGE T_1_w scans were acquired using imaging parameters based on the ADNI-3 protocol (Kruggel et al., 2010; Weiner et al., 2017) but without in-plane acceleration (TR/TI/TE = 2300/900/2.9 ms, Matrix = 256x240x192, Resolution = 1 mm isotropic, Scan Time = 9:14 min). This scan duration is longer than typically used today, where an in-plane acceleration factor of 2 is commonly employed to bring scan duration to a range of 5–6 min. However, we desired comparable baseline images without sparse sampling to not have other characteristics (e.g., g-factor-related non-uniform noise amplification) resulting from the effects of in-plane GRAPPA acceleration and reconstruction (Griswold et al., 2002).

Five CS protocols were tested, varying in the amount of under-sampling (CSx2, CSx4, CSx6, CSx8, CSx10), yielding scan times of 3:36, 1:51, 1:14, 0:58 and 0:47 min, respectively. A separate scan was required for each under-sampling value as the Research Application sequence did not allow for retrospective under-sampling in the inline reconstruction on the scanner’s reconstruction computer. As mentioned previously, beyond initial testing, CSx10 was not acquired. CS protocol parameters were matched to the ADNI-3 MPRAGE protocol whenever possible (TR/TI/TE = 2300/900/2.9 ms, Matrix = 256x240x192, Resolution = 1 mm isotropic). The turbo factor (number of phase encodings within one readout block after the inversion) was kept as close to 192 as possible for each CS under-sampling factor to match that of the MPRAGE protocol and to avoid differential degrees of T_1_-weighting and blurring. Additionally, all images were reconstructed employing the vendor’s PreScanNormalize feature to significantly reduce image intensity non-uniformity in three-dimensions that result from B_1_^+^/B_1_^−^ effects (Narayana et al., 1988; Vovk et al., 2007; Belaroussi et al., 2006). Each set of 4 CS images (CSx2, CSx4, CSx6, CSx8) was repeated 4 times within the same session. The order was counterbalanced across sets of CS scans. All CS images were first reconstructed using a recommended regularization parameter (Reg = 0.0003). Each CS T_1_w image was additionally retrospectively reconstructed at the scanner console using 4 additional regularization settings determined empirically (Reg = 0.0001, 0.0006, 0.0009, 0.0012). The entire process was then repeated within 7 days. Altogether, a total of 82 T_1_w images were collected per session (or 164 images per person).

### Image processing

Images were visually inspected for quality, as well as quantitatively assessed using the MRIQC toolbox (Esteban et al., 2017). Estimates of SNR of gray matter and white matter tissue, as well as image smoothness are presented here. We recognize the CS image reconstruction process can result in highly manipulated SNR values, however, we report the resulting metrics here in order to quantify the effect of under-sampling and reconstruction algorithms on the observed image quality. The MRIQC toolbox uses the FSL-FAST tool (Zhang et al., 2001) to perform basic gray and white matter segmentation. Here, SNR is defined as the mean signal in the tissue mask divided by the standard-deviation of the signal across the mask (mriqc.readthedocs.io/en/latest/iqms/t1w.html#mriqc.qc.anatomical.snr) (Esteban et al., 2017). This method could include inherent signal variation across the tissue due to uncorrected B_1_^+^/B_1_^−^ effects and proton-density variation, as well as image noise, resulting in lower SNR values than might be derived from other methods. However, it is easily calculated from magnitude-only DICOM images and captures the effect of noise in the tissue region, which manifests non-uniformly across the field of view (Esteban et al., 2017; Dietrich et al., 2007). MRIQC uses the AFNI program 3dFWHMx (afni.nimh.nih.gov/pub/dist/doc/manual/3dFWHM.pdf) to calculate the degree of image spatial smoothness (spatial correlation of voxels) as determined from the calculated full-width-half-maximum (FWHM) of the spatial distribution of the image intensity values (Forman et al., 1995). This parameter was recognized by Friedman et al. (2006) as an important metric in cross-scanner comparisons in multi-site studies. Lower values are better, higher values indicate a blurrier image. The original derivation has values in units of voxels (Forman et al., 1995), however they are commonly reported in units of mm (Friedman et al., 2006).

Estimates of brain structure were obtained using the Freesurfer v6.0 imaging analysis suite (surfer.nmr.mgh.harvard.edu). Details of this processing have been described previously (Dale et al., 1999; Fischl, 2012; Fischl and Dale, 2000; Fischl et al., 2002; Fischl et al., 1999; Fischl et al., 2004; Reuter et al., 2010; Ségonne et al., 2004; Buckner et al., 2004; Sled et al., 1998). All images were processed using the standard cross-sectional processing stream without scan-to-scan co-registration. Briefly, each image underwent brain extraction through a hybrid watershed/surface deformation procedure (Ségonne et al., 2004), the estimated total intracranial volume (eTIV) was calculated (which is directly proportional to the atlas scaling factor) (Buckner et al., 2004), and image intensity normalization (Sled et al., 1998). After preprocessing, images further underwent automated volume-based segmentation (Fischl et al., 2002; Fischl et al., 2004) to obtain structural brain estimates related to subcortical regions including the thalamus, caudate, putamen, pallidum, hippocampus, amygdala, and ventral diencephalon. Cortical surfaces were estimated by constructing a tessellated mesh across vertices (~150,000 vertices per hemisphere) separating pial from gray matter and gray from white matter tissue boundaries (Fischl and Dale, 2000). Cortical thickness was calculated as the shortest distance between these two pial/gray matter and gray/white matter boundaries. Regional cortical thickness was parcellated using the Desikan-Killaney atlas (Fischl et al., 1999; Desikan et al., 2006). Subcortical volumes (*n* = 14) and regional cortical thickness (*n* = 68) estimates were compared within and between protocols. T_1_w images and corresponding surface- and volume-based estimates were visualized using the freeview tool within the FreeSurfer imaging suite (surfer.nmr.mgh.harvard.edu/fswiki/FreeviewGuide).

### Statistical analysis

All statistical tests and plots of FreeSurfer morphometric estimates were carried out using R (r-project.org). Within-sequence agreement was evaluated by comparing the *test–retest repeatability* of estimates obtained using the same imaging protocol, within the same day, separately in each participant. Within-session agreement measures were averaged across two days. Results are displayed for the two participants separately. Between-sequence agreement was calculated as the Pearson correlation (R^2^) between the estimates obtained from CS vs. fully sampled MPRAGE imaging protocols within the same individual in the same session. That is, we asked the question of how much of the variation across measures estimated from a single MPRAGE scan could be accounted for by a single CS scan. Since at least two of each sequence type was acquired during each session (i.e., 2 MPRAGE T_1_w), this correlation was calculated twice within the same day, and twice again on the second day, resulting in four independent between-sequence comparisons in the same person. Between-session agreement measures of all four comparisons, and their correlation (R^2^) are displayed for the two participants.

Images were quantitatively compared by calculating the Structural Similarity Index (SSIM) (Wang et al., 2004), a commonly used metric that is based on the multiplication of terms and differences relating to contrast, luminance and the structure. Calculation was done between pairs of images—either the fully sampled MPRAGE to a CS-accelerated version, or between two reconstructions of the same under-sampled k-space where different regularization values were used. We used the SSIM3D tool as implemented in TensorFlow-MRI (mrphys.github.io/tensorflow-mri/api_docs/tfmri/image/ssim3d/), while the relevant images were co-registered using the FreeSurfer robust_register tool (Reuter et al., 2010). To further highlight the effect of regularization on image reconstruction, *fslmaths* from FSL toolbox (Jenkinson et al., 2012) was employed to take the absolute difference between images reconstructed using two adjacent regularization factor settings: |0.0001, 0.0003|, |0.0003, 0.0006|, |0.0006, 0.0009|, and |0.0009, 0.0012|. As the input k-space matrix is otherwise identical, the absolute difference between the reconstructed images captures the amount of information being introduced when varying the regularization parameter used during image reconstruction.

## Results

### Image quality

Visual inspection of raw T_1_w images acquired using a fully sampled MPRAGE and parameter-matched CS protocols did not reveal any major differences. Sagittal and coronal slices of raw T_1_w images across MPRAGE and CSx2, CSx4, CSx6, CSx8, and CSx10 scans are displayed in Figure 1 from a pilot participant. With greater under-sampling, the images appear more pixelated or “grainy” as lower signal and amplified noise reduce SNR non-uniformly. This effect can be seen most clearly when viewing subcortical structures and surrounding white matter tissue. The white arrows point to ringing artifacts commonly seen at CSx10 and occasionally at CSx8. As mentioned previously, CSx10 was not acquired after the first participant due to the presence of these artifacts.

**Figure 2:**
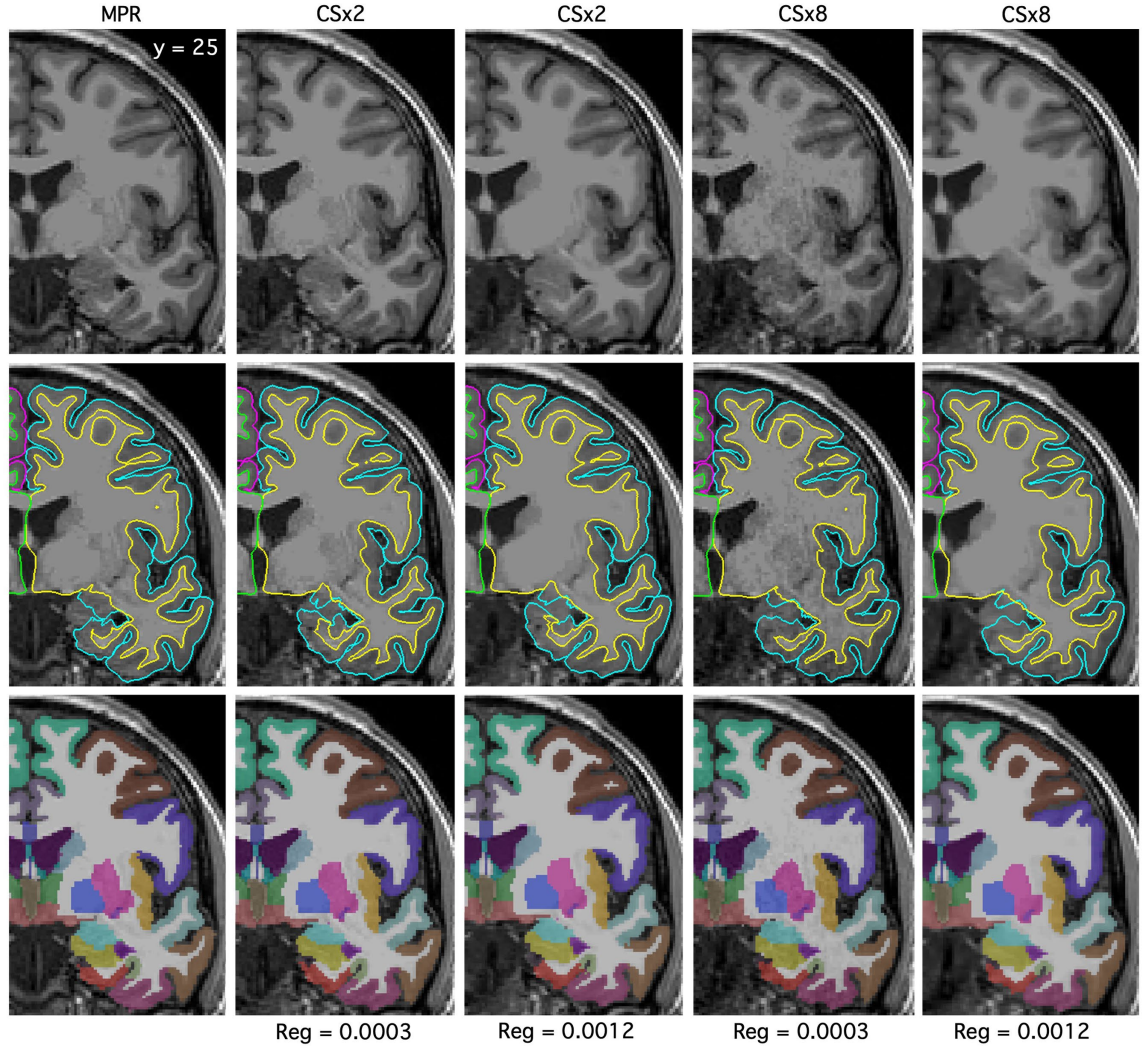
Example raw T_1_w images, surface-based and volume-based morphometric estimates are displayed for fully sampled (MPR), CSx2 and CSx8 MPRAGE protocols. Additionally, the CS images are displayed with default regularization (Reg = 0.0003) and retro-reconstructed with high regularization (Reg = 0.0012). As seen in Figure 1, images acquired with greater under-sampling (CSx8) have reduced Signal-to-Noise Ratio (SNR) compared to those at lower levels of under-sampling (CSx2). This noise-amplification can be controlled by choice in regularization factor, but at the potential cost of image SNR and smoothness. Surface-based estimates of the pial/gray matter boundaries are displayed in blue and the white matter/gray matter boundaries are displayed in yellow. Volume-based cortical parcellation and subcortical segmentation are shown in the third row. A legend of the color shadings used to denote cortical and subcortical regions in the third row is given in Supplementary Figure 3.

Though CS T_1_w images with greater under-sampling appear granular, this noise-amplification can be controlled by choice in regularization factor, but at the potential cost of image smoothness. Figure 2 shows raw T_1_w images, with resulting surface-based and volume-based estimates overlayed for the fully sampled MPRAGE and CSx2 and CSx8 protocols acquired in the same image set. CS images are shown when reconstructed with the default regularization setting (Reg = 0.0003) and with high regularization (Reg = 0.0012). The effect of regularization is seen most clearly in images acquired with greater under-sampling (CSx8). Surface-based estimates overlayed on the raw T_1_w image from each protocol are displayed in the middle row. The left temporal lobe pial/gray matter boundaries are displayed in blue and the white matter/gray matter boundaries are displayed in yellow. Finally, volume-based estimates are overlayed on the same raw T_1_w image and are shown in the third row. Note that these tracings are nearly identical at low (Reg = 0.0003) and high (Reg = 0.0012) levels of regularization, suggesting the regularization term has little effect on the calculations for surface-based or volume-based morphometric estimates, even though the appearance of certain subcortical structures may be impacted with high regularization.

**Figure 3:**
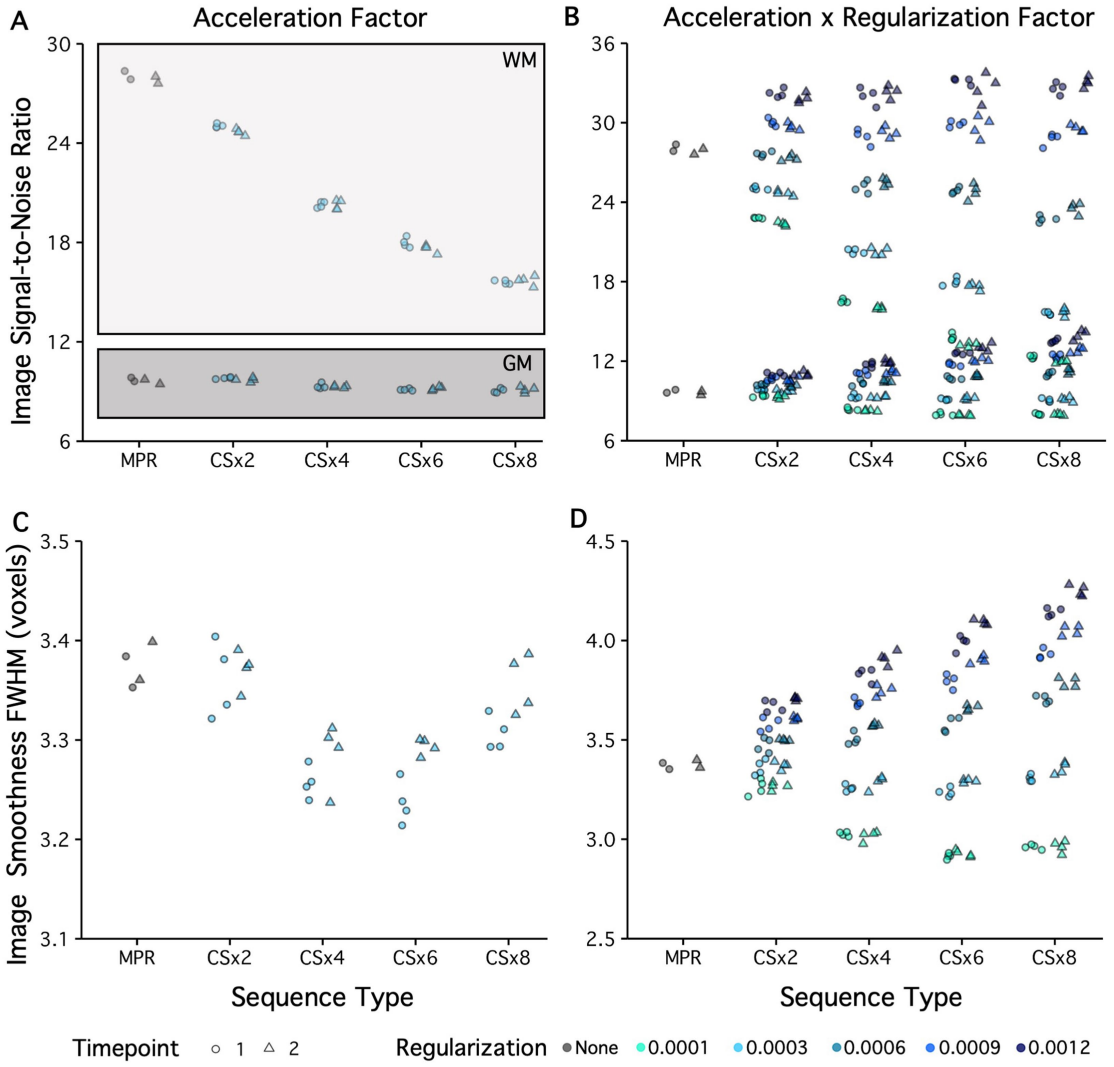
Tissue-class Signal-to-Noise Ratio (SNR) and image smoothness estimates for all scan types are displayed from one participant acquired across two different days. Shape denotes timepoint and color denotes the regularization factor used. **(A)** Gray matter and white matter SNR are estimated from fully sampled (MPR) and CS MPRAGE protocols (with default regularization Reg = 0.0003). Gray matter SNR was equivalent across all protocols. By contrast, white matter SNR was reduced in all images acquired using CS protocols compared to MPR. **(B)** Gray matter and white matter SNR can be further manipulated by regularization factor, in particular white matter SNR can be maintained at a level similar to the MPR acquisition. **(C)** Image smoothness, as estimated by the full-width-half-maximum (FWHM) kernel present, is displayed for the MPR and CS protocols (with default regularization Reg = 0.0003). Image smoothness was equivalent between MPR and CSx2 protocols, but then showed a parabolic relationship with CS factor: reduced at CSx4 and CSx6, but increased at CSx2 and CSx8. **(D)** Imaging smoothness can also be altered by choice of regularization in image reconstruction. Changes in regularization have a more pronounced effect at high levels of under-sampling and in some cases resulted in image smoothness that exceeded that of the fully sampled MPR image.

As a quantitative assessment of image quality, tissue-class SNR and image smoothness are displayed for all T_1_w images acquired across two sessions in one participant (Figure 3). Shape denotes timepoint (2 separate sessions) and color denotes the regularization factor used. Gray matter and white matter SNR are displayed for the fully sampled MPRAGE and variant CS protocols (reconstructed with default regularization) in the top left plot (see Figure 3A). Gray matter SNR was equivalent across all protocols. By contrast, white matter SNR was reduced in all CS T_1_w images compared to those acquired using the fully sampled MPRAGE protocol. White matter SNR appears directly proportional to the CS under-sampling factor, such that white matter SNR continued to decline as the CS under-sampling factor was increased. Gray matter and white matter SNR were both influenced by modifying the regularization factor (see Figure 3B). In particular, the white matter SNR seen in a CS image acquisition can be set to a similar value as seen in the fully sampled MPRAGE acquisition by tuning the regularization factor. Note at high levels of under-sampling (CSx8) and low regularization (Reg = 0.0001) white matter SNR is approaching that of gray matter SNR.

Image smoothness (or spatial correlation), as estimated by the FWHM of the spatial distribution of the image intensity values, is displayed for all CS and fully sampled MPRAGE T_1_w images acquired in one participant in Figure 3C. Image smoothness was more-or-less equivalent between the fully sampled MPRAGE and CSx2 protocols, but then showed a parabolic relationship with CS factor, such that image smoothness was reduced at CSx4 and CSx6 and increased at CSx8. Image smoothness is also altered by tuning the regularization parameter (see Figure 3D). Changes in regularization have a more pronounced effect on smoothness at high levels of under-sampling and regularization can be tuned to yield image smoothness of a similar value as seen in the fully sampled MPRAGE image.

### CS images show high within-sequence agreement

Within-sequence agreement was evaluated by comparing the *test–retest repeatability* of morphometric estimates obtained from T_1_w images acquired using the same protocol on the same day in the same participant; then averaged across days. Note in this small study, an estimate of reliability measured across many participants is not possible [see Elliott et al. (2023) for a traditional estimate of reliability for a CS under-sampled variant protocol where a large sample of participants were scanned across independent sessions]. The purpose here of measuring within-session repeatability is to have a metric that can quantify the effects of acceleration and reconstruction parameters.

**Figure 4:**
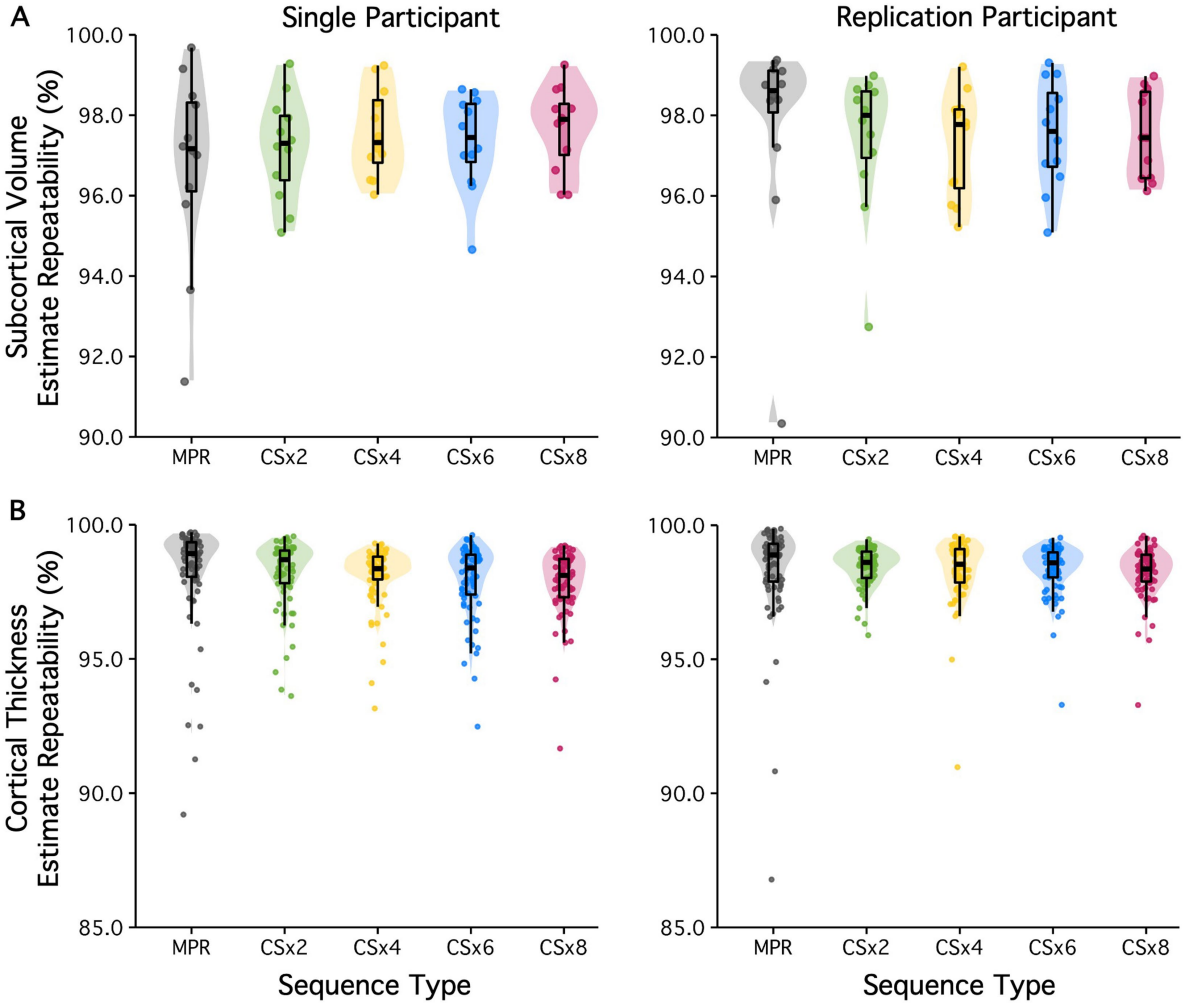
Subcortical volume and regional cortical thickness estimate agreement is presented for fully sampled (MPR) and CS MPRAGE protocols for two participants. Here, CS images were all reconstructed with default regularization (Reg = 0.0003). **(A)** Subcortical volume estimates showed high repeatability across all sequence types (>90%). **(B)** With the exception of the temporal poles, regional cortical thickness estimates showed high repeatability across all sequences (>90%). Most notably, estimates obtained using accelerated protocols showed comparable repeatability to those obtained from the 9 min fully sampled protocol. Color coding of the data points/clouds indicates different under-sampling levels. The estimate agreement values are tabulated with structure/region in Supplementary Table 1.

Figure 4 shows the within-session test–retest repeatability of all (A) subcortical volume and (B) regional cortical thickness estimates obtained from the fully sampled MPRAGE and CS under-sampled variant protocols (all with default regularization). Results are displayed for both participants. Subcortical volume estimates showed high agreement across all protocols (repeatability of all estimates were above 90%). Amygdala, pallidum, and thalamic volume estimates had the lowest agreement, while hippocampus, caudate and putamen volume estimates showed the highest repeatability in both participants. Regional cortical thickness estimates (except for the temporal pole) showed high agreement (> 90%) across all protocols. The temporal pole, frontal pole, transverse temporal cortex and entorhinal cortex estimates had the lowest agreement in both participants across all protocols. Most notably, estimates obtained using accelerated protocols showed comparable test–retest repeatability to those obtained from the 9-min fully sampled MPRAGE protocol. The repeatability values tabulated by structure are provided in Supplementary Table 1.

**Figure 5:**
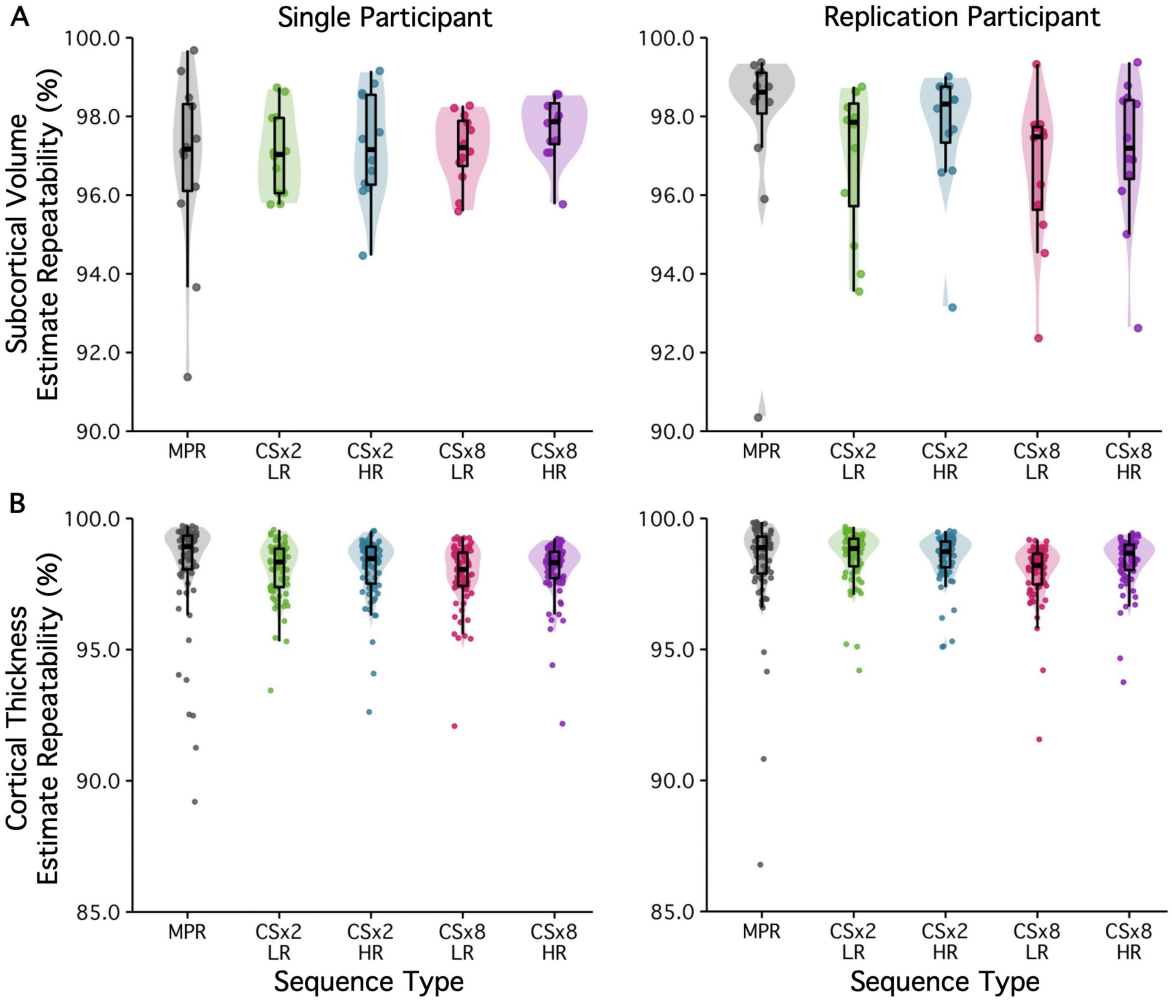
The effect of regularization on subcortical volume and regional cortical thickness estimate agreement is displayed for two participants. Results are displayed for the fully sampled (MPR) protocol, as well as CSx2 and CSx8 MPRAGE protocols, reconstructed with low (Reg = 0.0001) and high regularization (Reg = 0.0012). **(A)** Subcortical volume estimates showed high repeatability across all sequence types (>90%). The regularization factor appears to have little to no effect on subcortical volumes estimate repeatability, despite its impact on white-matter SNR. **(B)** Similarly, regional cortical thickness estimates showed high repeatability across most of the cortex. In all cases, the repeatability of the estimate had more to do with its spatial location than which CS under-sampling or regularization parameter was used. Color coding of the data points/clouds indicates different under-sampling levels. The estimate agreement values are tabulated with structure/region in Supplementary Table 2.

To test the effect of regularization in image reconstruction on estimate repeatability, estimates obtained from images acquired at low and high levels of under-sampling were compared when reconstructed with a low or high regularization factor. All CS-based estimates were compared to those obtained from the fully sampled MPRAGE protocol (see Figure 5). Subcortical volume estimates showed high within-sequence agreement across all protocols (R^2^ > 90%). Regions showing the lowest repeatability when comparing between low and high levels of CS under-sampling (i.e., amygdala, pallidum, and thalamic volume estimates) continued to be among the least repeatable at both low and high levels of regularization. Similarly, regions showing high agreement (i.e., hippocampus, caudate and putamen volume estimates) continued to show high repeatability at low and high levels of regularization. In fact, regularization appears to have little to no effect on subcortical volume estimates of test–retest repeatability, despite its impact on white-matter SNR. Regional cortical thickness estimates (except for the temporal pole) showed high repeatability (>90%) across all imaging protocols. As before, temporal pole, frontal pole, transverse temporal cortex and entorhinal cortical thickness estimates continue to show the lowest agreement across all protocols, including across all CS under-sampling factors and regularization factors tested. As with subcortical volumes estimates, the regularization factor had little to no effect on the test–retest repeatability of regional cortical thickness estimates. Again, the repeatability values tabulated by structure are provided in Supplementary Table 2.

### CS images show high between-sequence agreement

**Figure 6:**
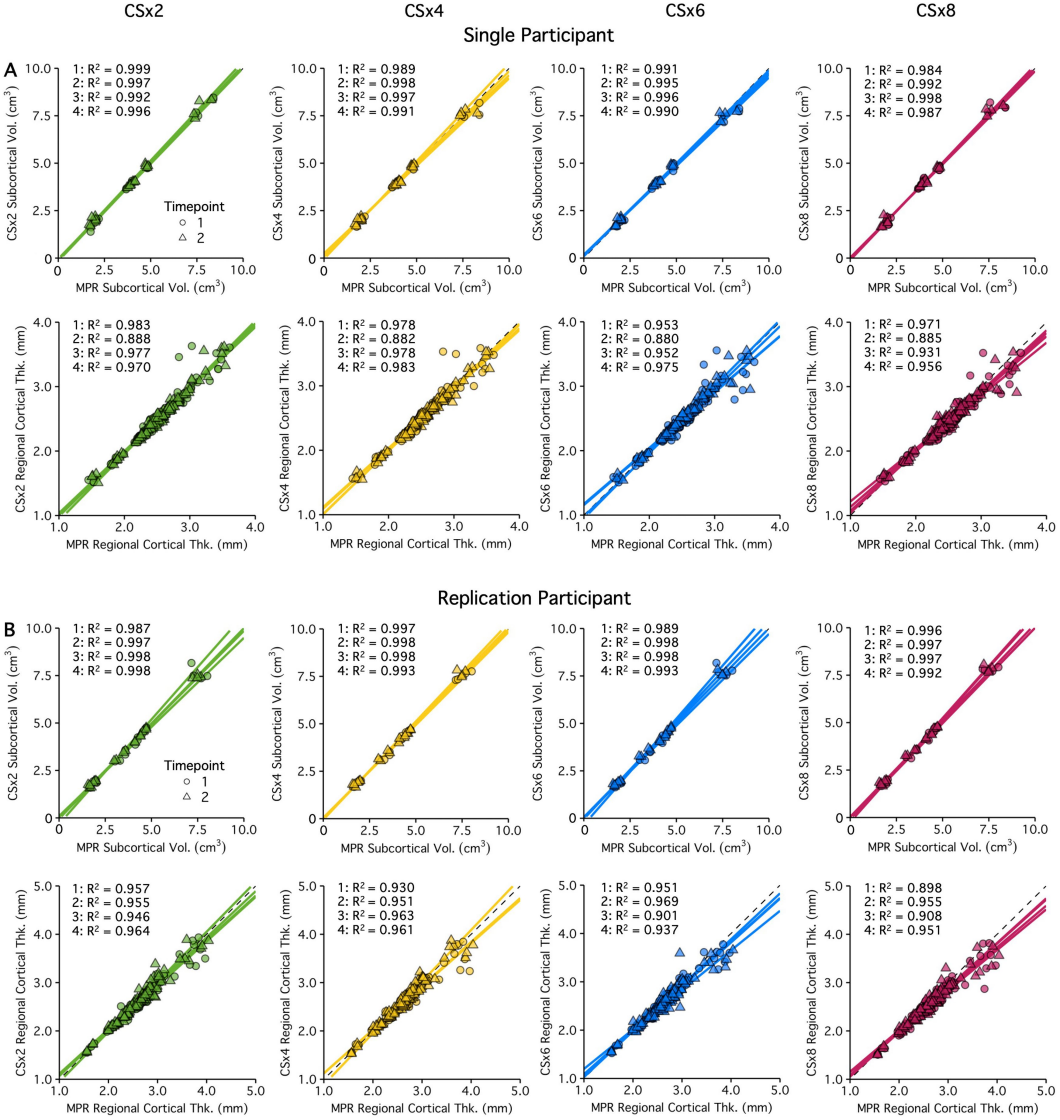
Between-sequence agreement of subcortical volume and regional cortical thickness estimates obtained from the fully sampled MPRAGE (MPR) protocol were compared to those from the CS protocols (all reconstructed with default regularization Reg = 0.0003) acquired within the same session in a single **(A)** and replication participant **(B)**. Each sequence type was acquired twice per session, and repeated during a second session, totaling four between-sequence comparisons. Shape denotes timepoint, and the correlation R^2^ of the four independent tests are presented here. Results are displayed for two participants. Color coding of the data points indicates different under-sampling levels. The volume and thickness values are tabulated by structure/region in Supplementary Table 3.

Between-sequence agreement was evaluated by comparing estimates from the fully sampled MPRAGE T_1_w image to the CS-accelerated image variants acquired within the same session. Figure 6 displays the between-sequence agreement of volume and surface-based estimates by comparing 4 separate sets of CS vs. fully sampled T_1_w images. Shape denotes timepoint, and the four between-sequence correlation R^2^ values are displayed on the top left of each plot. Across both participants, subcortical volume estimates showed excellent agreement (R^2^ ≥ 0.99) even at the highest levels of under-sampling. Collectively, regional cortical thickness estimates were also highly comparable between fully sampled and CS protocols (R^2^: 0.96–0.92). Between-sequence agreement was slightly reduced at higher levels of under-sampling (CSx8 vs. CSx2), as noted across both participants. However, we found that estimates obtained using the 58 s CSx8 protocols still showed excellent agreement (R^2^ > 90% in both participants) when compared to those obtained from the fully sampled 9 min MPRAGE protocol. The morphometric estimates plotted in Figure 6 are provided by structure in Supplementary Table 3.

**Figure 7:**
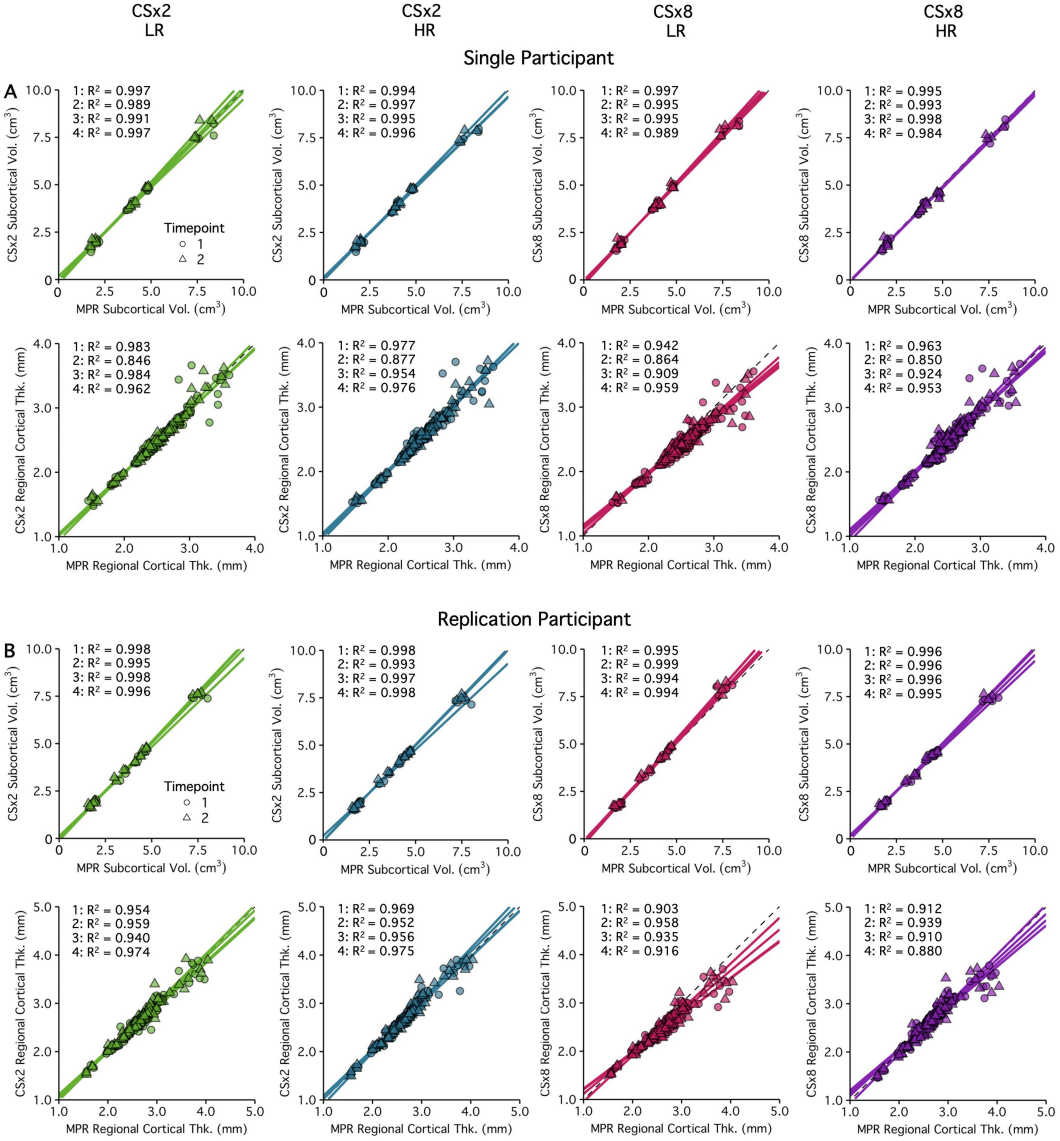
The effects of regularization on subcortical volume and regional cortical thickness estimate between-sequence agreement are displayed for a single **(A)** and replication participant **(B)**. Estimates obtained from the fully sampled MPRAGE (MPR) protocol were compared to those from the CSx2 and CSx8 protocols (reconstructed with low (Reg = 0.0001) and high regularization (Reg = 0.0012)). acquired within the same session. Each sequence type was acquired twice per session, and repeated during a second session, totaling four between-sequence comparisons. Shape denotes timepoint, and the correlation R^2^ of the four independent tests are presented here. Results are displayed for two participants. Color coding of the data points indicates different under-sampling levels. The volume and thickness values are tabulated by structure/region in Supplementary Table 4.

To test the effect of regularization in image reconstruction on between-sequence agreement, estimates obtained from images acquired at low and high levels of CS under-sampling were compared when reconstructed with a low or high regularization factor and to those obtained from the fully sampled MPRAGE protocol. As noted above, each sequence type was acquired at least 2 times per imaging session, allowing for a total of four between-sequence comparisons. Figure 7 displays the agreement between the fully sampled MPRAGE and CS under-sampled T_1_w images reconstructed with a low or high regularization factor. Subcortical volume estimates showed excellent agreement (R^2^ ≥ 0.99) regardless of the CS under-sampling factor or regularization factor used. Similarly, regional cortical thickness estimates were highly comparable between fully sampled and CS protocols, with only a minor reduction in estimate sensitivity seen at greater under-sampling (CSx8). Once again, regularization showed minimal impact on the agreement of morphometric estimates obtained using CS or fully sampled protocols. Again, the morphometric estimates plotted in Figure 7 are provided by structure in Supplementary Table 4.

### Structural similarity and effects of CS regularization on image reconstruction

Structural Similarity Indices (SSIM) were calculated between the fully sampled MPRAGE images in a session, and between one of those images and each accelerated scan at the different under-sampling values. Results are shown in Table 1 for each subject, along with the standard error of the mean from the multiple SSIM indices averaged for each under sampling value. SSIM values are high in all cases but do trend down as the degree of under-sampling increases. For both subjects, SSIM for repeated fully-sampled MPRAGE images was ~ 0.98, and ranging from ~ 0.98 for CSx2 down to ~ 0.96 for CSx8. This indicates that in comparison to the fully sampled image, the key structural and intensity features of the images are maintained with the under-sampling and CS reconstruction, despite the loss in SNR.

**Table 1:** Mean Structural Similarity Indices calculated between a fully sampled MPRAGE image (MPR) and compressed-sensing (CS) MPRAGE images with under-sampling factor x2, x4, x6 and x8 for both subjects, within each session.

Sequence type	No. of comparisons	Subject 01		Subject 02	
		SSIM	Std error	SSIM	Std error
MPR	2	0.9864	0.0018	0.9858	0.0003
CSx2	16	0.9801	0.0007	0.9824	0.0007
CSx4	16	0.9764	0.0006	0.9745	0.0008
CSx6	16	0.9726	0.0006	0.9703	0.0007
CSx8	16	0.9678	0.0007	0.9641	0.0010

The number of comparisons indicates the number of individual images acquired with each protocol compared to a single a fully sampled MPRAGE image from that session. SSIM values from each comparison were then averaged across the two sessions. CS images used the default regularization of 0.0003.

**Figure 8:**
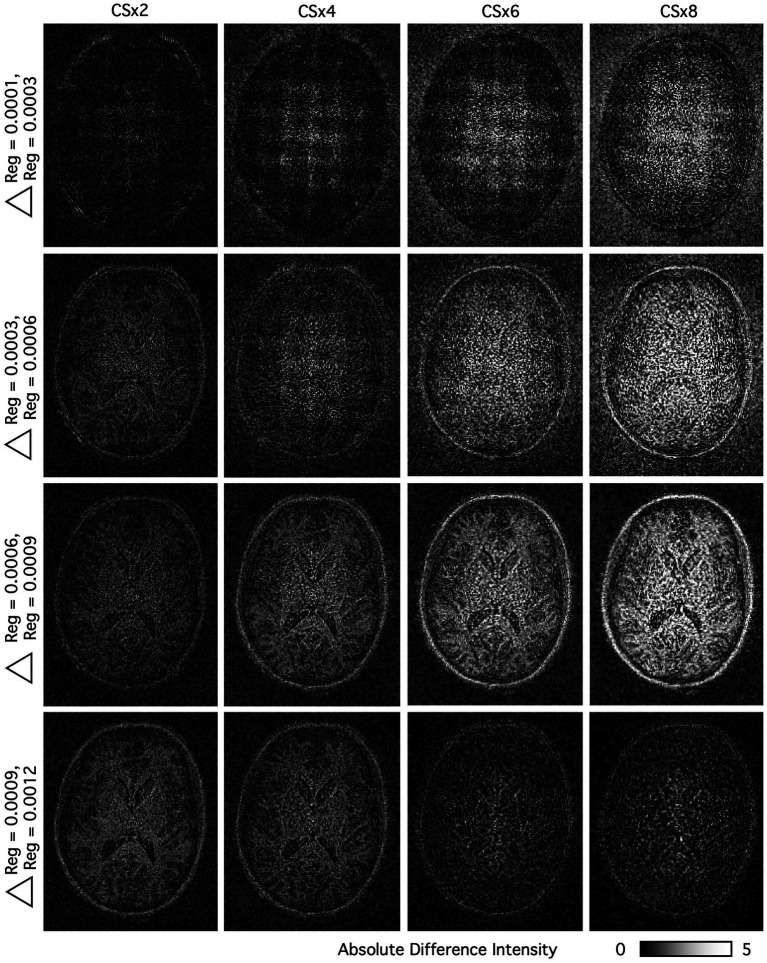
Effects of regularization on image reconstruction are shown in the axial plane. The absolute difference images were calculated from otherwise identical CS T_1_w images (varying only the regularization factor during the reconstruction process). At higher levels of under-sampling, more datapoints are extrapolated by the reconstruction algorithm. The images show the location and intensity at a pixel-level that is being introduced or modified by the image reconstruction algorithm when moving from a lower (Reg = 0.0001) to a higher (Reg = 0.0012) regularization value.

As a final evaluation of regularization on image reconstruction, absolute difference images were calculated from retro-reconstructed CS T_1_w images, using the identical k-space matrix as an input, and varying only the regularization factor during the reconstruction process. The images displayed in Figure 8 show the spatial location and change in intensity values introduced by the image reconstruction algorithm when moving from a lower (top) to a higher (bottom) regularization value. The goal of these figures was to highlight, for each acceleration level, at what regularization values only noise was being removed from the images and what values resulted in changes in the image structure, due either to smoothing or intensity variation. Image comparisons should only be made in the vertical columns, for each under-sampling value, as each column reflects differences in multiple reconstructions from a single k-space matrix. Supplementary Figure 1 shows the same set of absolute difference images in a coronal plane.

For CSx4 – CSx8, the change in regularization from 0.0001 to 0.0003 resulted only in noise removal, although there is an unusual grid-like pattern in the structure of the noise. We suspect this is due to the increase in regularization removing coefficients that contain image information in the wavelet domain. For CSx4, central noise removal remains the predominant effect of moving to Reg = 0.0006. While this is also true at CSx6 and CSx8, the well-defined structural detail of the scalp is also beginning to be impacted. Beyond Reg = 0.0006, the detailed structure of the brain is well-defined, with this effect more noticeable at the higher levels of under-sampling. By contrast, the CSx2 dataset shows little in the way of amplified noise removal at any regularization value, presumably due to the already high SNR in the images with limited under-sampling, while the brain structure also becomes more noticeable at higher regularization values.

To quantify these effects, SSIM indices were also calculated between images from the same k-space matrix but reconstructed with Reg. = 0.0001 and the higher regularization values. These indices, averaged across all images with the same under-sampling and regularization values, are shown in Table 2. Similarly to the qualitative interpretation of Figure 8, CSx2 images showed little change across all regularization values, SSIM ranging from ~ 0.99 for Reg. 0.0001 and 0.0003 to ~ 0.96 for Reg. 0.0001 and 0.0012. The change in SSIM between Reg. 0.0001–0.0003 and Reg. 0.0001–0.0012 grew monotonically with under-sampling, ranging from ~ 0.99 – ~ 0.91 for CSx4 to ~ 0.97 to ~ 0.87 for CSx8. These changes in SSIM match with the degree of change in each subtraction between images with different regularization values reconstructed from the same k-space matrix. Additionally, these can act as additional guidance against over-regularization in the CS reconstruction, highlighting for a particular under-sampling level, when a regularization change induces a larger variation in image quality than the difference between a fully sampled and under-sampled image.

**Table 2:** Mean Structural Similarity Indices calculated between the compressed-sensing (CS) MPRAGE images reconstructed with Reg. = 0.0001 and the same images reconstructed with higher regularization factors.

Acceleration factor (CS)	Regularization factor (Reg)	No. of comparisons	Subject 01		Subject 02	
			SSIM	Std error	SSIM	Std error
CSx2	0.0003	8	0.9965	0.0001	0.9919	0.0024
	0.0006	8	0.9874	0.0003	0.9829	0.0029
	0.0009	8	0.9763	0.0004	0.9692	0.0050
	0.0012	8	0.9661	0.0005	0.9591	0.0051
CSx4	0.0003	8	0.9874	0.0001	0.9839	0.0033
	0.0006	8	0.9581	0.0003	0.9551	0.0032
	0.0009	8	0.9350	0.0004	0.9316	0.0038
	0.0012	8	0.9199	0.0005	0.9151	0.0033
CSx6	0.0003	8	0.9803	0.0003	0.9771	0.0035
	0.0006	8	0.9318	0.0005	0.9330	0.0030
	0.0009	8	0.9032	0.0004	0.9012	0.0033
	0.0012	8	0.8912	0.0003	0.8840	0.0026
CSx8	0.0003	8	0.9770	0.0005	0.9673	0.0079
	0.0006	8	0.9157	0.0009	0.9224	0.0023
	0.0009	8	0.8839	0.0005	0.8889	0.0022
	0.0012	8	0.8739	0.0005	0.8720	0.0022

The results from four such comparisons per subject, per session were averaged across and within sessions for a total of eight comparisons.

## Discussion

While rapid CS protocols for T_1_w imaging show promise, to date it has been unclear whether the time savings they provide come at a cost. This study investigated the tradeoffs of time-saving CS-accelerated MPRAGE protocol parameter options and how the parameter choice impacts image quality, repeatability, and between-sequence agreement of the obtained structural estimates. Our results suggest that for most estimates, ultra-rapid CS imaging protocols can be adopted with little downside on morphometric analysis.

Image quality can be made largely comparable between CS and fully sampled MPRAGE protocols through an optimized combination of under-sampling and regularization during image reconstruction. While greater under-sampling does cause T_1_w images to appear more pixelated, especially in the center of the field-of-view, increasing the regularization factor can overcome the SNR deficit, while SSIM remains high regardless of under-sampling level or regularization. Higher under-sampling did not impact SNR in gray matter tissue but, by contrast, greatly and proportionally reduced the SNR in white matter tissue. SNR loss is to be expected due to the reduced number of RF pulses and readouts as a result of the under-sampling, as well as non-uniform noise-amplification in the reconstruction – phenomena also found in the use of GRAPPA in-plane acceleration (Griswold et al., 2002). Despite its effect on white matter SNR, tuning the regularization factor had little-to-no impact on the agreement of brain estimates acquired at the same under-sampling factor. Image smoothness was largely influenced by the regularization parameter and showed the largest modulating effects at high levels of CS under-sampling. However, caution must be exercised to avoid over-regularization if the area of interest is the white matter and subcortical structures within it. While the structural estimates from FreeSurfer remain consistent, Figure 2 shows that high regularization values can cause loss of contrast between the white matter and some sub-cortical structures, such as the pallidum and putamen in this case.

Within-sequence agreement was comparable across the fully sampled MPRAGE and accelerated CS imaging protocols. Across all imaging protocols, the structural estimates were stable. Consistent with our earlier work (Elliott et al., 2023), the present study suggests estimates obtained from the 72 s CSx6 protocol were just as repeatable as those obtained from the 9-min standard MPRAGE protocol. Notably, regions showing the lowest agreement were also among the smallest in size. These included the temporal pole, frontal pole, transverse temporal cortex and entorhinal cortex, which have previously been noted as having the least reliable morphometric values (Fischl et al., 2002; Fischl et al., 2004; Desikan et al., 2006). Again, modifying the regularization parameter had little to no effect on within-sequence agreement.

High between-sequence agreement was seen between estimates obtained from accelerated CS and fully sampled MPRAGE T_1_w images. Even when comparing high levels of under-sampling (CSx8) to the fully sampled MPRAGE imaging protocol, we found strong agreement in subcortical volume and regional cortical thickness estimates when collected in the same sessions. To some degree, this is not surprising, given that the GM SNR is retained regardless of under-sampling level; while the FreeSurfer segmentation process for each subcortical structure uses knowledge of the relative size and position with respect to other nearby structures (Fischl, 2012) which assume greater importance in the case of poor SNR or contrast. This is in line with our previous work on MPRAGE acceleration methods where we found brain morphometric estimates were fairly robust to reduced SNR (Nielsen et al., 2019; Mair et al., 2016; Mair et al., 2018). While the absolute value of a brain estimate may vary slightly between imaging protocols, the relative positioning of each estimate (i.e., the individual ranking order) was preserved. Varying the regularization factor also had minimal-to-no impact on estimate between-sequence agreement. While we note high between-sequence agreement, it is still not recommended to combine estimates obtained from different sequence types without correction because of shifts in absolute values.

Despite the mostly high agreement, a few regional cortical thickness estimates consistently diverged from the correlation lines in the top right of those plots (see Figures 6, 7). These regions included the temporal pole, frontal pole, transverse temporal cortex and entorhinal cortex. Not only are these regions among the smallest in surface area, they are also located where susceptibility-induced magnetic field gradients can cause problems with image distortion or dropout. Turning to visual inspection of the raw T_1_w images, we noted that this Research Application sequence yielded significant contrast variations presumably due to susceptibility artifacts in the medial orbitofrontal and inferior temporal gyri, that were present at all levels of CS under-sampling (Hanford et al., 2021), also see Supplementary Figure 2. Supplementary Figure 2 shows an example raw T_1_w image in a coronal slice, as well as a zoomed in portion of the left temporal lobe. The surface-based tracing from the fully sampled MPRAGE and the CSx6 T_1_w imaging protocols are displayed in blue and pink, respectively. Note how the fully sampled MPRAGE and CSx6 surface-based tracings diverge only in these areas of high susceptibility.

In later testing, we discovered the susceptibility artifacts seen in these frontal and temporal regions could be removed by altering the orientation in which the images were acquired (i.e., acquiring slices in coronal instead of sagittal orientation such that the non-cartesian trajectories in k_y_ and k_z_ were swapped). With this modification, local susceptibility-induced artifacts were less prominent. Therefore, we assume that these susceptibility effects depend on the trajectory chosen to sample k-space. The surface-based tracing of the experimental coronally-acquired CSx6 T_1_w scan is shown in yellow in Supplementary Figure 2. Finally, the bottom row of the figure displays all three surface-based estimates overlaid on the same MPRAGE T_1_w image. Note how similar these estimates/tracings appear across all other regions of the cortex. Ongoing work is focused on using the coronally-acquired CSx6 T_1_w protocol with further validation against a standard sagittally-acquired cartesian MPRAGE, as demonstrated in our recent studies with older adults (Elliott et al., 2023; Elliott et al., 2024).

Our study is not without limitations. First, we used a small number of participants – two for the main study—as our primary goal at the outset was to determine optimal regularization parameters for a given acceleration level. This was done using repeated reconstructions of the same k-space matrix; while multiple highly under-sampled scans were acquired from each subject in each session. The differences in SNR and smoothness between acceleration levels, along with the consistency of those values across days and the two subjects, indicate the robustness of our approach for this question. However, our participants had previous scanner experience and had exhibited little intra-scan motion in those sessions, nearly guaranteeing our data would be free from serious motion artifacts and confounds. Therefore, the estimates of repeatability and agreement we report are likely higher than what would be possible had we used a larger participant cohort including patients, older adults, and children, and the optimal protocol values we determined may not necessarily be suitable for children or older adults with or without cognitive impairment. To that end, we note the hBCD study in the USA has adopted more conservative CS under-sampling values for use with infants and young children (Dean et al., 2024) than we found optimal here. Moreover, the variance accounted for by our repeatability estimate was across measures acquired within the same session, not reliability as is often measured using variation between participants. However, in our prior work, we studied older individuals with neurodegeneration, who had more movement, and CS imaging protocols performed extremely well in a traditional assessment of between-session test–retest reliability (Elliott et al., 2023).

Second, our study was conducted using a 3 T Siemens MAGNETOM MRI scanner with a novel MPRAGE sequence with sparse sampling and a CS reconstruction developed as a Research Application sequence by the authors who are Siemens employees. It is difficult to know whether our testing of under-sampling and regularization factors will translate directly to other vendor CS imaging implementations or alternative implementations on a Siemens scanner. Indeed, as we showed above, image quality in high-susceptibility areas may be impacted by k-space trajectory, and as such, any other implementation of sparse sampling in k-space should be inspected for agreement with conventional imaging methods. Third, the reference fully sampled MPRAGE imaging protocol used in this study had a scan duration that is longer than typically used today. Instead, an in-plane acceleration factor of 2 is commonly employed to bring scan duration to the 5-min range. However, we opted for baseline images without any under-sampling to keep other characteristics constant between comparisons (e.g., g-factor-related non-uniform noise amplification) resulting from the effects of in-plane GRAPPA acceleration and reconstruction (Griswold et al., 2002).

In addition to the above, we note the potential for further advances in image acceleration and image quality from rapid acquisitions provided by the advances in Deep-Learning reconstruction approaches developed in recent years (Knoll et al., 2019; Zhu et al., 2018; Knoll et al., 2020; Yu et al., 2022) Rather than relying on a discrete mathematical transformation of the k-space data, Deep-Learning approaches acknowledge that low-level image features – often independent of the actual image content - can be learned from large data-sets. In addition, the incorporation of data-driven priors into the reconstruction can learn to remove under-sampling artifacts and handle low SNR even in the case of otherwise more conventional reconstruction approaches. Such an approach has shown the possibility for accelerating image acquisition further not by additional under-sampling but by modification of acquisition parameters, at the cost of SNR that would be considered unacceptable in standard reconstructions (Yu et al., 2025). Such methods will form a focus of future studies into image acquisition acceleration for brain morphometry and will likely yield faster scan times than those presented here.

Finally, we note that this publication focuses on the use of T_1_w images for brain morphometry. Our observations of comparable image quality between fully sampled and rapid imaging technique was based on quantitative image quality metrics and resulting structural estimates obtained from the images. While the estimate uncertainty from a single highly accelerated scan remains similar to that from a single fully-sampled scan, we note the ability to reduce those uncertainties by acquiring multiple rapid scans and averaging the morphometrics from each scan, as we have previously demonstrated (Nielsen et al., 2019; Elliott et al., 2024). This is a crucial step toward the goal of being able to accurately track neuro-degeneration. Given annual hippocampal atrophy rates are ~ 1–2% in cognitively unimpaired adults vs. 3–6% in those with Alzheimer’s Disease, measurement errors from a single scan on the order of 2–5% make it difficult to determine morphometric estimates with better accuracy than the change one is trying to measure. We have already shown the ability to reduce measurement error in certain morphometric estimates to below 1% by pooling results from 5–8 repeated rapid scans, which can now be acquired in the same time as 1–2 conventional scans without adding to patient burden (Elliott et al., 2024).

However, we note that with default regularization values in the reconstruction, the CS images do appear noisier than fully sampled scans, especially in central brain regions. On the other hand, over-regularization can wash-out central brain regions, resulting in loss of contrast between sub-cortical structures and the white matter around them. This may be problematic for uses that require visual reading of images. Certainly, the value of CS acceleration has been recognized in the clinic (Jaspan et al., 2015; Sartoretti et al., 2018; Vranic et al., 2019; Meister et al., 2023; Picchi et al., 2024) and for other neuroimaging applications (Duan et al., 2020; Trotier et al., 2022; Beaumont et al., 2023; Rowley et al., 2024), especially when participants may be un-cooperative (Dean et al., 2024; Arani et al., 2024). However, users who visually read the images or have other qualitative purposes for them may not draw the same general conclusions as we have, or may find more conservative levels of acceleration better-suited to their purpose.

In summary, ultra-rapid CS imaging protocols can provide T1w images with comparable quantitative quality metrics to those obtained using longer protocols. For most brain morphometry estimates, rapid, highly under-sampled CS imaging protocols can be adopted [see also (Elliott et al., 2023; Elliott et al., 2024)]. With the current sequence implementation, for a 1.0 mm isotropic T1w image, we recommend a regularization of 0.0003 for CSx2 and CSx4, while something slightly higher (between 0.0003–0.0006) may be better for CSx6, improving SNR without compromising image smoothness too much. Acceleration of CSx8 or greater in our implementation appears inadequate to provide artifact-free images currently.

## Data Availability

The datasets presented in this article are not readily available because IRB approval at the time of study did not include wording for consent by subjects to sharing of data outside of immediate institutions and collaborators. Requests to access the datasets should be directed to Ross W. Mair, rmair@fas.harvard.edu.

## Supplementary material

SUPPLEMENTARY FIGURE 1 Effects of regularization on image reconstruction are shown in the coronal plane. The absolute difference images were calculated from otherwise identical CS T1w images (varying only the regularization factor during the retro-reconstruction process). At higher levels of under-sampling, more datapoints are extrapolated by the reconstruction algorithm. The images show the location and intensity at a pixel-level that is being introduced or modified by the image reconstruction algorithm when moving from a lower (Reg = 0.0001) to a higher (Reg = 0.0012) regularization value.

SUPPLEMENTARY FIGURE 2 Imaging protocol surface-based tracings. The top row shows the raw image and surface tracing of the left temporal lobe using the fully sampled MPRAGE (MPR) imaging protocol. The second row shows the same slice and surface tracing acquired using the CSx6 MPRAGE imaging protocol. The third row shows the same slice and surface tracing from the coronally-acquired CSx6 MPRAGE imaging protocol. Switching to a coronal acquisition removed the artifact seen in the temporal lobe without introducing any new ones. Surface tracings from all the sequences: MPR, CSx6 Sag, and CSx6 Cor have been overlayed on the same MPR T1w image in the bottom row. Note how nearly identical these tracings appear across all other regions of the cortex, except the inferior temporal cortex.

SUPPLEMENTARY FIGURE 3 A legend of the color coding of subcortical structures and cortical regions used in the bottom row of Figure 2.

SUPPLEMENTARY TABLE 1 Subcortical volume and regional cortical thickness estimate agreement values for fully sampled (MPR) and CS MPRAGE protocols for two participants. This is a tabulation of the data plotted in Figure 4.

SUPPLEMENTARY TABLE 2 Subcortical volume and regional cortical thickness estimate agreement values for the fully sampled (MPR) protocol, as well as CSx2 and CSx8 MPRAGE protocols, reconstructed with low (Reg = 0.0001) and high regularization (Reg = 0.0012). This is a tabulation of the data plotted in Figure 5.

SUPPLEMENTARY TABLE 3 Subcortical volume and regional cortical thickness estimates obtained from the fully sampled MPRAGE (MPR) protocol and those from the CS protocols (all reconstructed with default regularization Reg = 0.0003) acquired within the same session. Subcortical volumes have units of mm^3^, thicknesses are in mm. This is a tabulation of the data plotted in Figure 6.

SUPPLEMENTARY TABLE 4 Subcortical volume and regional cortical thickness estimates obtained from the fully sampled MPRAGE (MPR) protocol and those from the CSx2 and CSx8 protocols [reconstructed with low (Reg = 0.0001) and high regularization (Reg = 851 0.0012)], acquired within the same session. Subcortical volumes have units of mm^3^, thicknesses are in mm. This is a tabulation of the data plotted in Figure 7.
