# Supplementary material for: Optimizing ultra-rapid compressed-sensing MPRAGE acquisitions for brain morphometry
Source: Front Neuroimaging. 2026 Jan 2;4:1718444. doi: 10.3389/fnimg.2025.1718444 (PMC12807958; doi:10.3389/fnimg.2025.1718444)
Supplement: Supplementary file 1 [file Data_Sheet_1.PDF]

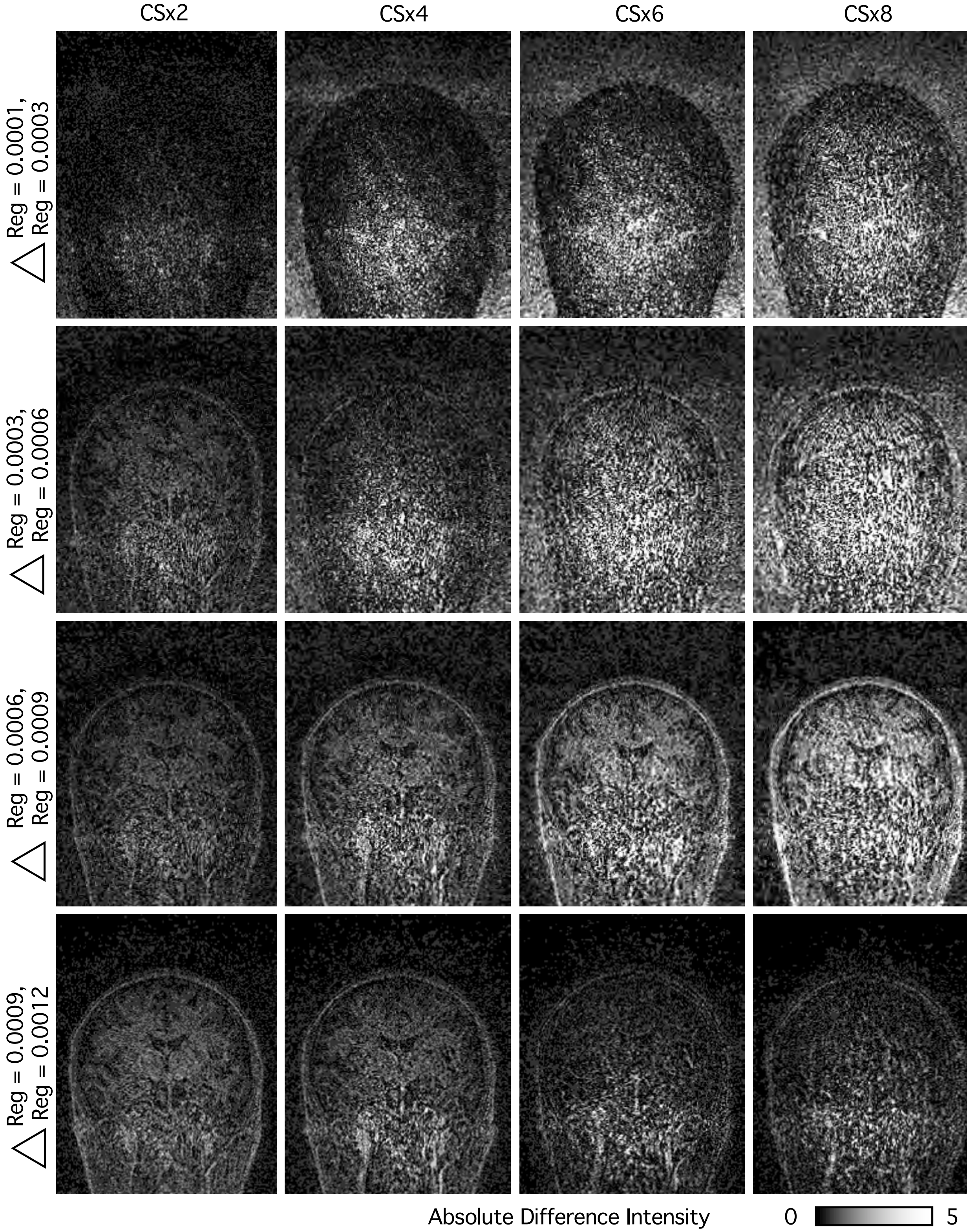

Supplemental Figure 1

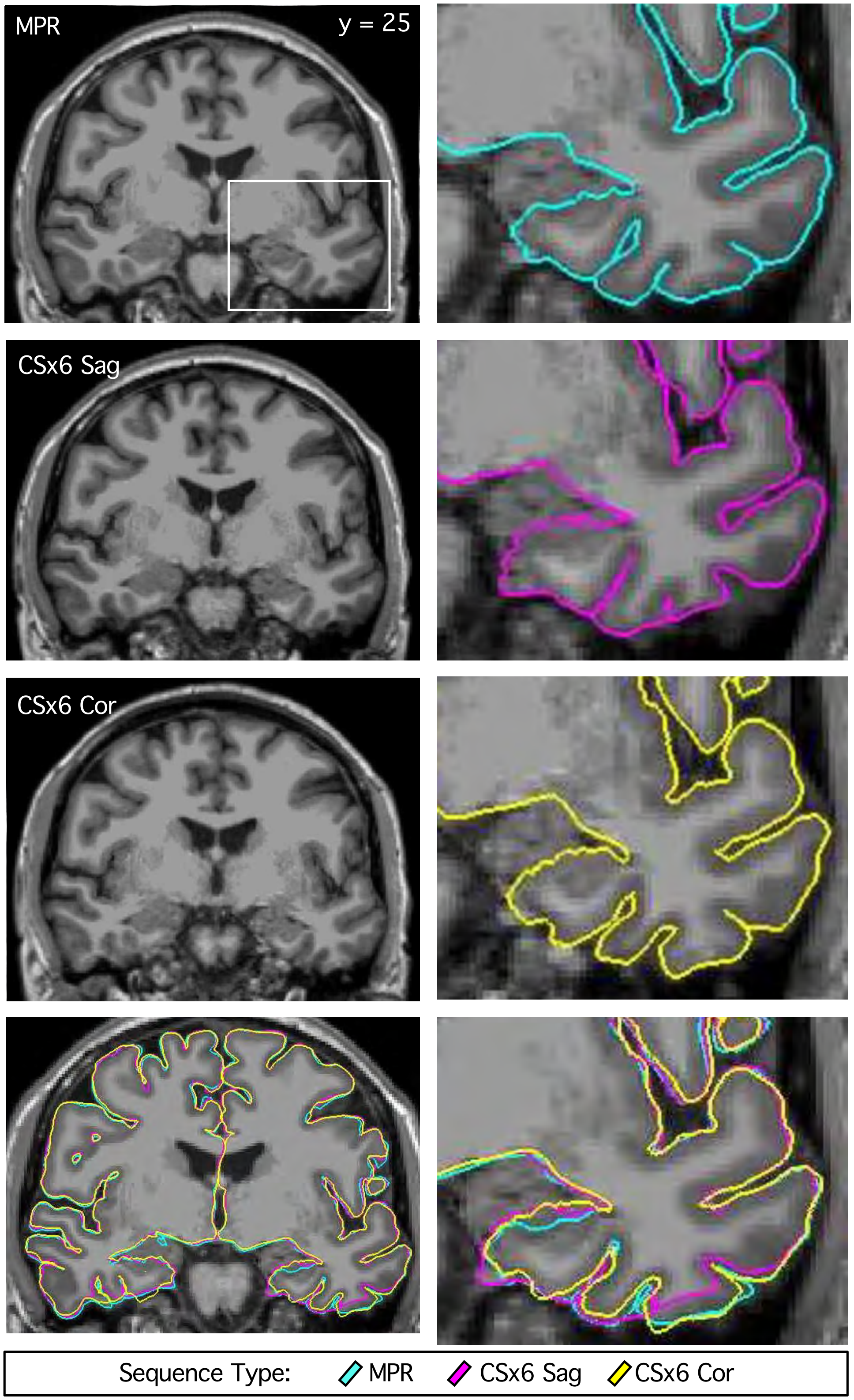

Supplemental Figure 2

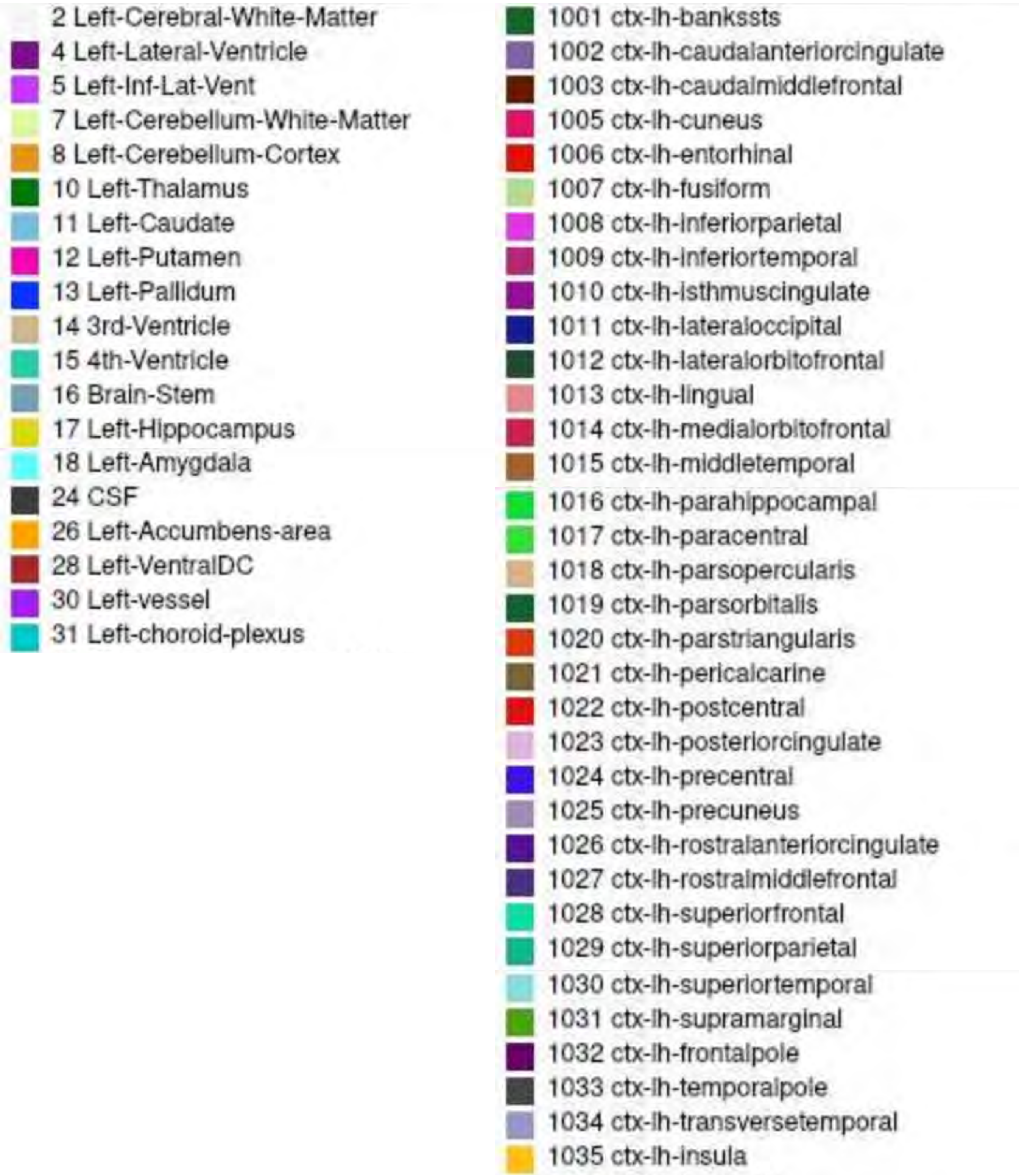

Supplementary Figure 3

		Subject01										Subject02									
	Protocol	MPR		CSx2		CSx4		CSx6		CSx8		MPR		CSx2		CSx4		CSx6		CSx8	
	Regularization	NA		0.0003		0.0003		0.0003		0.0003		NA		0.0003		0.0003		0.0003		0.0003	
		MEAN	Std Error	MEAN	Std Error	MEAN	Std Error	MEAN	Std Error	MEAN	Std Error	MEAN	Std Error	MEAN	Std Error	MEAN	Std Error	MEAN	Std Error	MEAN	Std Error
thalamus	Left	95.90	0.58	95.73	0.51	95.23	0.54	95.96	0.48	97.45	0.31	91.38	1.18	95.08	0.61	97.17	0.33	97.00	0.35	98.15	0.22
thalamus	Right	98.78	0.17	97.87	0.25	97.82	0.25	96.48	0.41	96.45	0.41	97.11	0.41	98.13	0.22	98.59	0.17	98.57	0.17	98.69	0.16
putamen	Left	98.36	0.23	97.52	0.29	98.18	0.21	98.16	0.22	98.56	0.17	99.15	0.12	99.28	0.08	99.24	0.09	98.36	0.19	97.79	0.26
putamen	Right	98.76	0.18	98.75	0.14	98.02	0.22	97.38	0.32	98.78	0.14	99.68	0.05	98.67	0.16	99.15	0.10	98.64	0.17	99.25	0.09
pallidum	Left	98.39	0.25	97.08	0.36	95.77	0.49	96.87	0.37	96.88	0.38	97.43	0.38	95.43	0.54	97.04	0.36	96.35	0.44	96.02	0.47
pallidum	Right	99.30	0.11	96.54	0.39	97.72	0.27	96.80	0.37	96.12	0.45	96.21	0.57	97.58	0.29	97.48	0.30	96.24	0.45	96.63	0.38
amygdala	Left	97.20	0.42	92.75	0.87	95.69	0.50	97.83	0.27	96.31	0.43	97.22	0.40	96.51	0.41	96.02	0.50	94.66	0.60	96.02	0.49
amygdala	Right	90.35	1.30	98.58	0.17	96.36	0.45	95.09	0.56	96.43	0.41	95.79	0.57	96.01	0.46	96.39	0.42	98.26	0.21	97.14	0.36
hippocampus	Left	98.48	0.22	98.13	0.22	98.67	0.16	99.31	0.08	98.98	0.12	98.26	0.25	97.15	0.35	96.96	0.37	97.17	0.32	97.86	0.25
hippocampus	Right	99.37	0.09	98.64	0.17	96.33	0.46	99.03	0.11	97.44	0.29	97.01	0.43	97.93	0.24	97.93	0.25	98.09	0.22	97.93	0.27
caudate	Left	99.15	0.12	98.98	0.12	98.14	0.23	98.41	0.19	98.34	0.19	93.66	0.89	97.38	0.32	98.30	0.19	97.02	0.34	98.16	0.21
caudate	Right	99.10	0.13	98.38	0.18	99.21	0.10	99.01	0.12	98.67	0.16	98.48	0.22	97.22	0.35	96.37	0.41	97.73	0.26	98.64	0.16
bankssts	Left	99.30	0.04	99.15	0.04	98.14	0.09	98.26	0.08	98.64	0.06	98.07	0.11	98.93	0.05	99.28	0.04	99.30	0.03	98.12	0.09
bankssts	Right	97.97	0.12	98.90	0.05	98.40	0.08	97.70	0.12	98.45	0.08	98.68	0.08	99.09	0.04	98.10	0.09	98.19	0.09	99.02	0.05
caudalanteriorcingulate	Left	99.15	0.05	98.31	0.08	97.97	0.10	97.59	0.11	97.80	0.11	97.18	0.18	96.24	0.18	96.36	0.17	96.98	0.15	97.60	0.12
caudalanteriorcingulate	Right	98.09	0.12	97.24	0.13	97.77	0.11	99.28	0.03	97.61	0.12	98.34	0.11	98.29	0.09	99.00	0.05	96.51	0.17	97.26	0.13
caudalmiddlefrontal	Left	99.23	0.05	98.75	0.06	99.00	0.05	98.87	0.05	98.90	0.05	98.87	0.07	99.40	0.03	98.22	0.08	98.45	0.08	97.87	0.11
caudalmiddlefrontal	Right	99.81	0.01	98.76	0.06	98.98	0.05	99.14	0.04	98.53	0.07	99.20	0.05	98.77	0.06	98.94	0.05	97.90	0.10	98.42	0.08
cuneus	Left	98.90	0.07	98.67	0.06	97.20	0.14	97.07	0.14	98.77	0.06	99.68	0.02	98.20	0.09	98.21	0.09	98.07	0.10	98.83	0.06
cuneus	Right	97.94	0.12	98.05	0.09	99.05	0.04	98.67	0.06	98.72	0.06	98.05	0.12	99.45	0.03	98.52	0.07	97.62	0.11	97.68	0.11
entorhinal	Left	94.15	0.38	96.32	0.18	90.98	0.47	93.30	0.32	93.29	0.32	96.31	0.26	93.62	0.31	93.15	0.33	95.41	0.22	94.24	0.27
entorhinal	Right	94.90	0.35	97.61	0.11	97.62	0.12	98.43	0.08	95.71	0.21	94.04	0.40	93.85	0.30	94.10	0.29	95.52	0.21	91.66	0.41
frontalpole	Left	99.02	0.06	99.21	0.04	98.06	0.09	98.59	0.07	98.30	0.08	91.26	0.55	95.45	0.23	94.88	0.25	94.27	0.28	97.22	0.14
frontalpole	Right	97.73	0.13	98.56	0.07	97.67	0.11	96.77	0.17	97.27	0.13	99.09	0.06	94.51	0.26	96.26	0.18	94.82	0.25	97.90	0.11
fusiform	Left	99.18	0.05	99.07	0.05	98.94	0.05	98.82	0.06	97.94	0.10	98.98	0.06	98.86	0.06	98.36	0.08	99.01	0.05	98.14	0.09
fusiform	Right	98.39	0.10	98.61	0.07	98.95	0.05	98.68	0.07	97.92	0.10	98.48	0.09	99.05	0.04	98.24	0.09	99.18	0.04	98.61	0.07
inferiorparietal	Left	98.98	0.06	98.52	0.08	99.11	0.04	98.60	0.07	99.51	0.03	99.21	0.05	98.92	0.05	98.81	0.06	98.39	0.08	97.17	0.14
inferiorparietal	Right	98.72	0.08	99.08	0.05	98.44	0.08	98.41	0.09	98.33	0.08	99.46	0.03	98.36	0.08	98.18	0.09	98.62	0.07	99.00	0.05
inferiortemporal	Left	98.10	0.12	97.96	0.10	97.97	0.10	98.39	0.08	98.39	0.08	99.08	0.06	98.16	0.09	98.89	0.05	98.39	0.08	97.56	0.12
inferiortemporal	Right	99.13	0.05	98.52	0.07	99.01	0.05	99.19	0.04	97.65	0.11	98.58	0.09	98.90	0.05	98.43	0.07	98.47	0.08	98.87	0.06
insula	left	97.79	0.13	97.79	0.11	94.99	0.24	97.13	0.14	97.92	0.10	97.52	0.15	96.69	0.17	98.15	0.09	96.44	0.17	96.64	0.16
insula	right	97.74	0.14	97.67	0.11	97.40	0.13	96.59	0.16	97.23	0.13	99.49	0.03	98.89	0.05	98.04	0.10	97.35	0.13	98.10	0.09
isthmuscingulate	Left	97.59	0.14	96.53	0.17	97.40	0.13	98.05	0.10	97.59	0.12	98.41	0.10	98.18	0.09	98.38	0.08	97.58	0.11	98.52	0.07
isthmuscingulate	Right	96.85	0.19	96.91	0.15	97.83	0.11	97.68	0.11	96.24	0.19	96.56	0.21	97.67	0.12	96.32	0.18	97.44	0.12	95.60	0.21
lateraloccipital	Left	99.75	0.02	98.68	0.06	98.89	0.05	98.63	0.07	98.86	0.05	99.32	0.04	98.97	0.05	98.81	0.06	98.71	0.07	98.08	0.09
lateraloccipital	Right	98.89	0.07	99.14	0.04	99.08	0.05	98.08	0.09	98.05	0.09	99.52	0.03	98.78	0.06	98.72	0.06	99.43	0.03	98.68	0.07
lateralorbitofrontal	Left	97.17	0.18	98.86	0.06	97.42	0.12	99.20	0.04	97.98	0.10	98.76	0.08	97.68	0.11	98.25	0.08	98.79	0.06	97.63	0.12
lateralorbitofrontal	Right	96.95	0.19	97.80	0.11	97.06	0.15	98.19	0.09	97.59	0.12	98.14	0.11	98.03	0.09	97.39	0.13	97.69	0.11	97.30	0.13
lingual	Left	98.95	0.06	98.99	0.05	98.63	0.07	98.01	0.10	98.66	0.07	99.29	0.04	99.11	0.04	98.84	0.06	98.23	0.09	98.97	0.05
lingual	Right	98.18	0.11	98.12	0.09	99.04	0.05	97.39	0.13	98.48	0.07	99.35	0.04	99.01	0.05	98.74	0.06	98.97	0.05	98.69	0.07
medialorbitofrontal	Left	98.50	0.09	98.03	0.09	97.69	0.11	98.10	0.10	98.82	0.06	97.91	0.14	98.25	0.08	98.18	0.09	96.36	0.18	96.03	0.20
medialorbitofrontal	Right	98.51	0.09	98.34	0.08	98.08	0.09	98.22	0.09	98.67	0.07	99.37	0.04	96.26	0.19	96.94	0.15	97.26	0.13	97.09	0.14
middletemporal	Left	99.80	0.01	98.72	0.06	99.20	0.04	98.78	0.06	99.61	0.02	99.18	0.05	99.12	0.04	98.82	0.05	99.08	0.04	97.66	0.11
middletemporal	Right	99.00	0.06	99.15	0.04	98.99	0.05	98.53	0.07	98.49	0.07	99.33	0.04	98.09	0.09	98.58	0.07	98.88	0.05	98.78	0.06
paracentral	Left	97.88	0.13	98.12	0.09	98.34	0.08	98.31	0.08	97.96	0.10	99.24	0.05	98.89	0.05	98.65	0.07	98.29	0.08	97.94	0.10
paracentral	Right	99.29	0.04	98.49	0.07	99.14	0.04	98.71	0.06	98.30	0.08	98.95	0.07	99.04	0.04	98.07	0.09	99.04	0.05	98.63	0.07
parahippocampal	Left	99.19	0.05	97.93	0.11	98.33	0.09	97.27	0.14	98.16	0.09	97.16	0.17	97.50	0.12	97.82	0.11	96.03	0.20	97.07	0.14
parahippocampal	Right	98.54	0.09	98.68	0.06	98.48	0.07	98.82	0.06	98.38	0.08	98.90	0.07	97.80	0.10	98.61	0.07	98.72	0.06	97.64	0.11
parsopercularis	Left	98.82	0.07	98.59	0.07	98.40	0.08	98.85	0.06	98.52	0.07	98.53	0.09	98.75	0.06	98.29	0.09	97.98	0.10	98.32	0.08
parsopercularis	Right	99.31	0.04	99.12	0.04	98.98	0.05	99.10	0.04	98.95	0.06	98.81	0.07	98.98	0.05	98.34	0.08	98.71	0.06	98.03	0.10
parsorbitalis	Left	97.68	0.14	98.70	0.06	98.08	0.09	98.59	0.07	97.37	0.13	97.86	0.13	98.06	0.09	98.24	0.09	97.06	0.14	96.76	0.15
parsorbitalis	Right	99.82	0.01	98.44	0.07	97.57	0.11	97.51	0.12	96.57	0.16	99.14	0.05	97.04	0.14	96.63	0.17	98.71	0.06	98.19	0.09
parstriangularis	Left	98.84	0.07	98.61	0.06	98.53	0.07	98.69	0.06	98.13	0.10	98.34	0.10	98.83	0.06	97.62	0.11	97.05	0.14	97.28	0.14
parstriangularis	Right	99.41	0.04	98.49	0.07	98.23	0.09	98.23	0.09	98.34	0.08	99.35	0.04	97.24	0.14	97.94	0.10	98.33	0.08	98.77	0.06
pericalcarine	Left	96.92	0.17	98.54	0.07	96.71	0.16	95.89	0.20	98.25	0.09	99.71	0.02	98.50	0.07	98.84	0.06	98.87	0.05	98.14	0.09
pericalcarine	Right	99.02	0.06	95.90	0.20	96.61	0.16	97.25	0.13	98.36	0.08	97.95	0.12	98.57	0.07	98.05	0.09	97.79	0.11	97.61	0.12
postcentral	Left	99.87	0.01	99.09	0.04	99.58	0.02	99.12	0.04	98.88	0.05	98.64	0.08	99.16	0.04	98.36	0.08	99.16	0.04	99.12	0.04
postcentral	Right	98.81	0.07	99.21	0.04	99.31	0.03	99.00	0.05	98.95	0.05	99.16	0.05	99.53	0.02	99.00	0.05	98.80	0.06	98.79	0.06
posteriorcingulate	Left	98.58	0.09	97.92	0.10	99.57	0.02	98.32	0.08	97.97	0.10	97.78	0.14	98.11	0.09	98.46	0.07	97.78	0.11	97.70	0.11
posteriorcingulate	Right	97.98	0.12	97.99	0.10	98.56	0.07	98.16	0.10	97.21	0.13	98.95	0.06	98.50	0.07	97.47	0.13	97.41	0.13	97.66	0.11
precentral	Left	99.54	0.03	99.30	0.03	99.27	0.04	98.62	0.07	99.10	0.04	99.65	0.02	99.57	0.02	98.80	0.06	99.61	0.02	99.07	0.05
precentral	Right	99.52	0.03	98.83	0.06	99.22	0.04	99.41	0.03	98.93	0.05	99.30	0.04	98.95	0.05	98.96	0.05	99.46	0.03	98.64	0.07
precuneus	Left	99.55	0.03	98.62	0.07	99.36	0.03	99.04	0.04	99.16	0.04	99.23	0.05	99.16	0.04	99.09	0.04	97.99	0.10	99.22	0.04
precuneus	Right	99.08	0.06	98.58	0.07	99.32	0.03	99.02	0.05	99.03	0.05	99.35	0.04	99.10	0.05	98.87	0.06	98.85	0.06	98.43	0.08
rostralanteriorcingulate	Left	97.27	0.17	97.92	0.10	97.97	0.10	98.61	0.07	97.84	0.10	97.26	0.17	96.69	0.15	98.08	0.09	97.84	0.10	95.93	0.20
rostralanteriorcingulate	Right	96.59	0.21	97.56	0.12	97.19	0.14	97.26	0.13	98.35	0.09	92.48	0.48	96.67	0.16	97.37	0.14	95.69	0.20	97.29	0.14
rostralmiddlefrontal	Left	99.73	0.02	98.76	0.06	98.94	0.05	98.86	0.06	99.01	0.05	99.37	0.04	98.37	0.08	98.40	0.08	97.39	0.12	97.76	0.11
rostralmiddlefrontal	Right	98.89	0.07	99.07	0.04	99.11	0.04	99.15	0.04	99.25	0.04	98.36	0.10	97.88	0.10	97.32	0.13	98.64	0.07	98.74	0.06
superiorfrontal	Left	99.29	0.04	99.02	0.05	99.30	0.04	98.96	0.05	99.31	0.04	99.63	0.02	99.48	0.03	98.70	0.06	98.92	0.05	98.33	0.08
superiorfrontal	Right	99.42	0.04	99.16	0.04	99.16	0.04	99.08	0.04	98.90	0.05	99.47	0.03	98.97	0.05	98.48	0.08	99.12	0.04	99.13	0.04
superiorparietal	Left	98.97	0.06	99.12	0.04	99.08	0.04	99.06	0.05	99.08	0.04	99.47	0.03	98.66	0.07	98.46	0.07	98.70	0.06	98.24	0.08
superiorparietal	Right	98.86	0.07	98.43	0.08	99.57	0.02	98.83	0.06	98.22	0.09	99.34	0.04	99.09	0.05	99.07	0.05	98.74	0.06	99.19	0.04
superiortemporal	Left	99.54	0.03	99.04	0.05	99.47	0.03	99.54	0.02	99.50	0.02	98.46	0.09	98.80	0.06	98.74	0.06	99.08	0.04	98.21	0.09
superiortemporal	Right	99.56	0.03	98.70	0.06	99.34	0.03	98.94	0.05	98.50	0.07	99.50	0.03	98.73	0.06	98.52	0.07	98.94	0.05	98.47	0.07
supramarginal	Left	99.36	0.04	99.00	0.05	99.44	0.03	99.24	0.04	98.90	0.05	98.18	0.11	99.13	0.04	99.30	0.03	98.93	0.05	98.78	0.06
supramarginal	Right	99.63	0.02	99.48	0.02	98.88	0.05	98.39	0.08	98.60	0.07	99.72	0.02	99.36	0.03	98.86	0.06	98.69	0.06	98.85	0.06
temporalpole	Left	86.79	0.76	97.76	0.11	97.06	0.14	98.33	0.08	95.94	0.19	92.53	0.46	97.75	0.11	98.18	0.09	96.92	0.15	95.65	0.23
temporalpole	Right	90.82	0.52	97.40	0.12	98.36	0.08	97.27	0.13	97.24	0.14	89.20	0.63	96.79	0.15	95.54	0.21	92.48	0.38	96.68	0.16
transversetemporal	Left	96.69	0.20	98.64	0.06	97.61	0.12	97.74	0.11	97.89	0.10	95.36	0.28	98.38	0.08	97.55	0.12	95.20	0.23	96.55	0.17
transversetemporal	Right	97.59	0.15	98.40	0.08	98.33	0.08	99.08	0.05	97.66	0.11	93.84	0.37	95.03	0.24	97.16	0.14	98.64	0.06	97.13	0.14

Supplementary Table 1

	Protocol<br>Regularization	Subject01<br>MPR<br>NA		CSx2<br>0.0001 (LR)		CSx2<br>0.0012 (HR)		CSx8<br>0.0001 (LR)		CSx8<br>0.0012 (HR)		Subject02<br>MPR<br>NA		CSx2<br>0.0001 (LR)		CSx2<br>0.0012 (HR)		CSx8<br>0.0001 (LR)		CSx8<br>0.0012 (HR)	
		MEAN	Std Error	MEAN	Std Error	MEAN	Std Error	MEAN	Std Error	MEAN	Std Error	MEAN	Std Error	MEAN	Std Error	MEAN	Std Error	MEAN	Std Error	MEAN	Std Error
thalamus	Left	95.90	0.58	94.71	0.64	96.58	0.44	96.27	0.44	95.01	0.58	91.38	1.18	96.05	0.50	96.18	0.48	97.65	0.27	97.90	0.26
thalamus	Right	98.78	0.17	98.62	0.18	98.77	0.14	95.76	0.51	96.90	0.37	97.11	0.41	97.08	0.36	97.60	0.28	95.79	0.49	98.02	0.25
putamen	Left	98.36	0.23	97.98	0.24	98.76	0.15	97.79	0.29	98.31	0.19	99.15	0.12	98.63	0.16	99.16	0.10	98.03	0.22	98.56	0.17
putamen	Right	98.76	0.18	98.23	0.21	98.75	0.14	97.46	0.30	96.51	0.44	99.68	0.05	98.73	0.15	98.59	0.16	98.21	0.21	98.57	0.16
pallidum	Left	98.39	0.25	93.99	0.72	96.62	0.40	97.71	0.30	97.46	0.29	97.43	0.38	95.77	0.51	96.62	0.39	96.47	0.41	95.77	0.49
pallidum	Right	99.30	0.11	96.06	0.45	98.42	0.19	94.53	0.67	92.62	0.85	96.21	0.57	96.68	0.40	97.43	0.30	95.59	0.53	98.54	0.18
amygdala	Left	97.20	0.42	93.55	0.75	93.14	0.79	92.36	0.94	96.11	0.47	97.22	0.40	95.76	0.49	94.46	0.66	96.83	0.37	97.08	0.33
amygdala	Right	90.35	1.30	97.19	0.32	97.57	0.28	95.25	0.59	96.93	0.37	95.79	0.57	96.05	0.46	96.11	0.46	96.95	0.37	97.37	0.31
hippocampus	Left	98.48	0.22	97.79	0.27	98.43	0.18	97.60	0.28	99.38	0.07	98.26	0.25	97.96	0.24	96.89	0.36	98.27	0.20	97.83	0.25
hippocampus	Right	99.37	0.09	97.91	0.25	97.67	0.28	97.51	0.28	98.39	0.20	97.01	0.43	97.98	0.24	98.83	0.13	97.31	0.33	98.27	0.20
caudate	Left	99.15	0.12	98.75	0.15	98.20	0.21	99.33	0.08	98.47	0.18	93.66	0.89	97.12	0.34	96.29	0.43	97.11	0.35	97.39	0.30
caudate	Right	99.10	0.13	98.64	0.16	99.01	0.12	97.80	0.26	98.78	0.14	98.48	0.22	96.98	0.37	98.54	0.17	97.84	0.25	97.08	0.35
bankssts	Left	99.30	0.04	99.03	0.05	98.76	0.07	97.14	0.14	98.19	0.09	98.07	0.11	98.56	0.07	99.02	0.05	98.66	0.06	99.20	0.04
bankssts	Right	97.97	0.12	98.79	0.06	98.61	0.07	98.48	0.07	98.92	0.05	98.68	0.08	97.49	0.13	98.48	0.07	97.77	0.10	97.54	0.12
caudalanteriorcingulate	Left	99.15	0.05	98.81	0.06	99.31	0.03	98.42	0.08	98.00	0.10	97.18	0.18	97.21	0.13	96.53	0.18	97.87	0.10	97.24	0.13
caudalanteriorcingulate	Right	98.09	0.12	98.14	0.09	98.00	0.10	98.42	0.08	97.00	0.14	98.34	0.11	98.73	0.06	97.45	0.12	97.61	0.12	98.40	0.08
caudalmiddlefrontal	Left	99.23	0.05	99.37	0.03	99.02	0.05	98.00	0.10	97.93	0.10	98.87	0.07	98.45	0.07	98.93	0.05	98.76	0.06	98.90	0.05
caudalmiddlefrontal	Right	99.81	0.01	99.57	0.02	98.40	0.08	97.53	0.12	98.87	0.05	99.20	0.05	98.37	0.08	98.99	0.05	97.55	0.12	98.27	0.08
cuneus	Left	98.90	0.07	98.44	0.08	99.00	0.05	98.35	0.08	98.70	0.06	99.68	0.02	98.79	0.06	98.14	0.09	98.71	0.06	98.84	0.06
cuneus	Right	97.94	0.12	98.51	0.07	98.32	0.08	96.80	0.16	98.46	0.08	98.05	0.12	98.32	0.09	98.47	0.07	97.94	0.11	98.31	0.08
entorhinal	Left	94.15	0.38	94.20	0.29	95.31	0.22	95.80	0.20	93.75	0.30	96.31	0.26	95.45	0.21	92.62	0.37	92.09	0.37	94.41	0.28
entorhinal	Right	94.90	0.35	95.11	0.25	95.10	0.24	94.21	0.27	97.23	0.14	94.04	0.40	93.44	0.32	96.87	0.15	95.41	0.23	96.81	0.16
frontalpole	Left	99.02	0.06	97.75	0.11	97.86	0.10	97.91	0.10	97.76	0.11	91.26	0.55	96.09	0.19	96.55	0.18	95.44	0.22	96.34	0.18
frontalpole	Right	97.73	0.13	98.85	0.06	97.96	0.10	96.82	0.15	97.68	0.11	99.09	0.06	95.32	0.22	94.09	0.29	96.05	0.19	95.78	0.20
fusiform	Left	99.18	0.05	99.26	0.04	99.19	0.04	98.30	0.08	98.77	0.06	98.98	0.06	98.40	0.08	98.84	0.06	98.95	0.05	98.43	0.08
fusiform	Right	98.39	0.10	99.22	0.04	99.16	0.04	97.24	0.13	99.04	0.05	98.48	0.09	99.17	0.04	99.53	0.02	98.62	0.07	99.06	0.04
inferiorparietal	Left	98.98	0.06	98.65	0.07	98.97	0.05	98.11	0.09	98.31	0.08	99.21	0.05	98.08	0.09	99.20	0.04	98.51	0.07	98.57	0.07
inferiorparietal	Right	98.72	0.08	99.24	0.04	98.68	0.06	98.58	0.07	98.95	0.05	99.46	0.03	98.91	0.05	98.12	0.09	97.82	0.10	98.13	0.09
inferiortemporal	Left	98.10	0.12	99.07	0.05	98.50	0.07	98.42	0.08	98.71	0.06	99.08	0.06	98.71	0.06	97.92	0.11	98.26	0.09	98.22	0.08
inferiortemporal	Right	99.13	0.05	98.49	0.07	98.69	0.06	97.88	0.10	98.96	0.05	98.58	0.09	98.36	0.08	98.31	0.08	98.10	0.09	98.98	0.05
insula	left	97.79	0.13	95.20	0.24	95.12	0.25	98.64	0.06	97.05	0.14	97.52	0.15	96.88	0.16	97.20	0.14	97.41	0.13	96.13	0.19
insula	right	97.74	0.14	97.97	0.10	96.50	0.16	97.00	0.15	96.39	0.17	99.49	0.03	98.06	0.09	98.27	0.08	97.42	0.13	97.92	0.10
isthmuscingulate	Left	97.59	0.14	97.24	0.14	98.59	0.07	96.76	0.16	97.51	0.12	98.41	0.10	98.54	0.07	98.85	0.06	97.04	0.15	97.80	0.11
isthmuscingulate	Right	96.85	0.19	97.67	0.11	98.45	0.07	97.53	0.12	94.66	0.25	96.56	0.21	98.16	0.09	97.35	0.13	97.91	0.10	97.53	0.12
lateraloccipital	Left	99.75	0.02	98.93	0.05	99.27	0.04	98.66	0.07	99.14	0.04	99.32	0.04	99.30	0.04	98.87	0.05	98.58	0.07	99.00	0.05
lateraloccipital	Right	98.89	0.07	99.38	0.03	99.20	0.04	98.69	0.07	99.20	0.04	99.52	0.03	98.72	0.06	98.59	0.08	98.88	0.05	99.04	0.05
lateralorbitofrontal	Left	97.17	0.18	98.77	0.06	98.12	0.09	98.72	0.06	98.41	0.08	98.76	0.08	96.67	0.16	98.47	0.07	98.37	0.08	98.32	0.08
lateralorbitofrontal	Right	96.95	0.19	97.22	0.13	97.70	0.11	97.39	0.13	99.44	0.03	98.14	0.11	97.95	0.10	97.59	0.12	96.50	0.17	98.59	0.07
lingual	Left	98.95	0.06	98.79	0.06	99.09	0.04	98.32	0.08	99.10	0.04	99.29	0.04	99.38	0.03	99.17	0.04	99.02	0.05	98.38	0.08
lingual	Right	98.18	0.11	98.04	0.10	98.58	0.07	97.28	0.13	98.79	0.06	99.35	0.04	98.56	0.07	98.37	0.08	97.43	0.13	97.55	0.12
medialorbitofrontal	Left	98.50	0.09	99.14	0.04	98.18	0.09	99.19	0.04	98.12	0.09	97.91	0.14	97.25	0.14	97.45	0.12	96.76	0.15	97.47	0.12
medialorbitofrontal	Right	98.51	0.09	99.00	0.05	99.23	0.04	96.63	0.16	98.24	0.09	99.37	0.04	98.05	0.10	97.74	0.12	96.25	0.18	98.06	0.09
middletemporal	Left	99.80	0.01	99.13	0.04	98.76	0.06	98.94	0.05	98.67	0.06	99.18	0.05	98.88	0.05	98.76	0.06	98.59	0.07	98.52	0.07
middletemporal	Right	99.00	0.06	98.99	0.05	99.18	0.04	98.47	0.08	98.46	0.07	99.33	0.04	98.09	0.09	98.68	0.07	97.65	0.11	98.39	0.08
paracentral	Left	97.88	0.13	98.62	0.07	98.68	0.07	98.02	0.10	98.72	0.06	99.24	0.05	98.89	0.05	99.00	0.05	98.43	0.08	98.84	0.06
paracentral	Right	99.29	0.04	99.20	0.04	99.03	0.05	98.19	0.09	98.26	0.09	98.95	0.07	98.69	0.06	98.78	0.06	99.29	0.03	98.44	0.09
parahippocampal	Left	99.19	0.05	97.08	0.15	97.97	0.10	97.11	0.14	98.72	0.06	97.16	0.17	97.38	0.13	98.42	0.08	97.65	0.12	98.47	0.08
parahippocampal	Right	98.54	0.09	98.27	0.08	99.17	0.04	98.04	0.10	99.04	0.05	98.90	0.07	98.03	0.10	98.67	0.07	97.29	0.13	98.30	0.08
parsopercularis	Left	98.82	0.07	99.45	0.03	98.85	0.05	98.13	0.09	98.78	0.06	98.53	0.09	98.84	0.06	98.27	0.08	98.38	0.08	97.72	0.11
parsopercularis	Right	99.31	0.04	99.35	0.03	98.72	0.06	97.46	0.13	98.53	0.07	98.81	0.07	98.10	0.09	98.95	0.05	97.45	0.12	98.65	0.07
parsorbitalis	Left	97.68	0.14	98.68	0.07	99.02	0.05	97.71	0.11	98.43	0.08	97.86	0.13	96.57	0.16	98.43	0.08	97.15	0.14	97.81	0.11
parsorbitalis	Right	99.82	0.01	98.48	0.07	97.61	0.12	96.22	0.19	98.42	0.08	99.14	0.05	97.44	0.13	96.30	0.17	97.59	0.11	97.56	0.12
parstriangularis	Left	98.84	0.07	99.22	0.04	98.83	0.06	98.15	0.09	98.91	0.05	98.34	0.10	97.04	0.14	98.74	0.06	98.20	0.09	97.78	0.11
parstriangularis	Right	99.41	0.04	98.67	0.06	99.02	0.06	97.35	0.13	98.55	0.07	99.35	0.04	97.64	0.11	97.11	0.15	97.74	0.11	98.16	0.09
pericalcarine	Left	96.92	0.17	97.90	0.10	97.38	0.13	98.13	0.09	97.85	0.11	99.71	0.02	98.24	0.09	97.29	0.13	97.83	0.11	98.22	0.09
pericalcarine	Right	99.02	0.06	97.14	0.13	98.15	0.10	97.63	0.11	99.04	0.05	97.95	0.12	97.71	0.11	98.45	0.08	98.03	0.10	98.63	0.07
postcentral	Left	99.87	0.01	99.52	0.02	99.53	0.02	99.16	0.04	99.29	0.03	98.64	0.08	99.30	0.04	98.94	0.05	99.08	0.04	98.57	0.07
postcentral	Right	98.81	0.07	99.18	0.04	98.78	0.06	98.77	0.06	99.40	0.03	99.16	0.05	99.57	0.02	98.95	0.05	98.87	0.06	98.80	0.06
posteriorcingulate	Left	98.58	0.09	97.56	0.12	98.59	0.07	98.23	0.09	97.42	0.12	97.78	0.14	96.65	0.17	98.19	0.09	97.62	0.11	98.24	0.10
posteriorcingulate	Right	97.98	0.12	98.32	0.08	97.84	0.10	97.92	0.10	97.99	0.10	98.95	0.06	98.88	0.06	98.77	0.06	98.05	0.10	98.31	0.08
precentral	Left	99.54	0.03	99.57	0.02	99.27	0.04	98.67	0.06	99.09	0.05	99.65	0.02	99.24	0.04	99.13	0.04	99.22	0.04	98.73	0.06
precentral	Right	99.52	0.03	99.44	0.03	99.24	0.04	98.85	0.06	99.03	0.05	99.30	0.04	99.23	0.04	99.11	0.04	98.84	0.05	98.91	0.05
precuneus	Left	99.55	0.03	98.72	0.06	98.84	0.06	98.57	0.07	98.68	0.06	99.23	0.05	98.85	0.06	98.90	0.06	98.30	0.08	98.19	0.09
precuneus	Right	99.08	0.06	99.21	0.04	98.39	0.08	98.92	0.05	99.29	0.04	99.35	0.04	99.04	0.05	98.81	0.06	99.21	0.04	98.73	0.06
rostralanteriorcingulate	Left	97.27	0.17	97.93	0.10	97.85	0.10	98.85	0.06	97.65	0.12	97.26	0.17	97.13	0.14	96.31	0.19	97.36	0.13	97.96	0.11
rostralanteriorcingulate	Right	96.59	0.21	98.86	0.06	97.59	0.12	98.12	0.09	96.71	0.15	92.48	0.48	97.37	0.13	95.29	0.23	95.52	0.22	96.35	0.18
rostralmiddlefrontal	Left	99.73	0.02	99.07	0.04	98.58	0.07	98.79	0.06	99.01	0.05	99.37	0.04	97.69	0.11	97.90	0.10	98.77	0.06	97.59	0.12
rostralmiddlefrontal	Right	98.89	0.07	99.58	0.02	99.07	0.05	98.35	0.08	99.20	0.04	98.36	0.10	97.24	0.13	97.14	0.14	98.06	0.09	97.73	0.11
superiorfrontal	Left	99.29	0.04	99.09	0.04	98.84	0.05	98.75	0.06	98.69	0.06	99.63	0.02	98.80	0.06	99.52	0.02	98.67	0.06	98.99	0.05
superiorfrontal	Right	99.42	0.04	99.34	0.03	99.11	0.05	98.61	0.07	99.07	0.04	99.47	0.03	98.69	0.07	98.68	0.06	98.29	0.08	98.64	0.07
superiorparietal	Left	98.97	0.06	98.93	0.05	99.12	0.04	98.62	0.07	98.70	0.06	99.47	0.03	98.77	0.06	98.44	0.08	98.56	0.07	98.90	0.05
superiorparietal	Right	98.86	0.07	99.53	0.02	98.13	0.09	98.29	0.08	98.38	0.08	99.34	0.04	98.93	0.05	98.62	0.07	99.19	0.04	98.40	0.07
superiortemporal	Left	99.54	0.03	99.30	0.04	99.49	0.02	98.91	0.05	99.02	0.05	98.46	0.09	99.00	0.05	99.12	0.04	98.51	0.07	99.14	0.04
superiortemporal	Right	99.56	0.03	99.06	0.05	98.94	0.05	98.81	0.06	98.87	0.05	99.50	0.03	98.63	0.07	98.60	0.07	98.95	0.05	98.92	0.06
supramarginal	Left	99.36	0.04	99.02	0.05	99.35	0.03	98.78	0.06	98.20	0.08	98.18	0.11	98.14	0.09	98.96	0.05	99.26	0.04	98.64	0.06
supramarginal	Right	99.63	0.02	99.40	0.03	99.17	0.04	98.12	0.10	98.70	0.06	99.72	0.02	99.44	0.03	99.05	0.05	98.77	0.06	98.75	0.06
temporalpole	Left	86.79	0.76	98.07	0.09	96.20	0.19	91.57	0.44	96.63	0.16	92.53	0.46	97.39	0.12	97.49	0.12	95.59	0.21	92.18	0.37
temporalpole	Right	90.82	0.52	97.29	0.14	97.86	0.11	97.43	0.12	98.25	0.09	89.20	0.63	96.97	0.15	96.79	0.15	96.12	0.19	96.10	0.19
transversetemporal	Left	96.69	0.20	97.67	0.11	98.32	0.08	98.56	0.08	97.68	0.12	95.36	0.28	96.67	0.16	97.73	0.11	97.62	0.11	97.63	0.12
transversetemporal	Right	97.59	0.15	98.75	0.06	99.47	0.03	96.87	0.14	98.31	0.08	93.84	0.37	96.82	0.17	96.95	0.15	96.76	0.16	96.74	0.16

Supplementary Table 2

		Subject01										Subject02									
	Protocol	MPR		CSx2		CSx4		CSx6		CSx8		MPR		CSx2		CSx4		CSx6		CSx8	
	Regularization	NA		0.0003		0.0003		0.0003		0.0003		NA		0.0003		0.0003		0.0003		0.0003	
		MEAN	Std Error	MEAN	Std Error	MEAN	Std Error	MEAN	Std Error	MEAN	Std Error	MEAN	Std Error	MEAN	Std Error	MEAN	Std Error	MEAN	Std Error	MEAN	Std Error
amygdala	Left	1733.7	23.6	1671.5	43.2	1678.8	21.8	1672.5	10.5	1645.6	17.9	1701.7	18.9	1633.7	17.8	1702.4	20.4	1697.0	28.0	1693.3	19.6
amygdala	Right	2000.4	78.4	2125.6	15.7	2136.5	27.4	2116.1	27.8	2138.8	22.7	1653.6	30.7	1739.9	21.2	1721.4	20.5	1790.4	12.6	1757.1	15.5
caudate	Left	3713.1	21.2	3697.5	16.6	3769.3	23.6	3742.2	25.4	3722.1	17.8	3099.5	71.4	3053.3	24.1	3105.4	13.9	3169.8	27.6	3208.9	17.3
caudate	Right	3876.5	18.5	3841.3	17.4	3863.4	9.2	3880.4	13.0	3866.8	15.0	3561.0	25.7	3517.3	29.6	3485.1	33.1	3586.4	24.0	3553.4	12.7
hippocampus	Left	4034.5	24.7	4028.4	22.1	4054.1	15.1	4040.0	7.4	4100.6	12.1	4142.8	36.4	4046.4	34.5	4117.1	35.0	4103.1	34.0	4135.0	27.2
hippocampus	Right	4064.7	51.5	4062.3	16.2	4075.5	43.2	4063.8	18.8	4106.5	37.0	4377.3	45.8	4319.2	25.9	4365.1	30.5	4355.6	24.1	4397.7	28.2
pallidum	Left	1979.4	15.9	1945.6	16.2	1988.9	24.9	1964.3	16.8	1897.3	18.6	1987.0	18.8	1960.7	24.8	2010.1	16.5	1972.7	22.3	1929.1	21.1
pallidum	Right	2046.3	13.4	2031.1	18.9	2005.6	13.6	2016.8	20.6	1861.8	26.6	1889.5	22.8	1921.7	16.5	1956.2	14.3	1927.4	20.6	1824.2	20.2
putamen	Left	4810.1	35.0	4893.0	32.6	4890.5	26.5	4896.1	28.2	4821.6	19.1	4546.7	14.3	4557.3	12.2	4517.4	14.1	4510.9	26.9	4609.6	27.4
putamen	Right	4791.7	24.6	4804.9	24.0	4714.3	25.9	4749.0	39.7	4718.8	19.0	4708.5	6.7	4684.7	18.4	4672.8	11.9	4722.4	21.2	4763.8	10.0
thalamus	Left	8173.7	182.6	8149.9	105.7	7913.3	100.3	7942.0	87.8	8012.2	72.9	7520.5	201.1	7710.3	125.5	7672.7	67.0	7758.9	73.0	7939.9	40.6
thalamus	Right	7441.4	48.4	7459.7	44.8	7567.6	46.6	7445.5	69.7	7818.1	86.2	7484.7	84.8	7485.7	37.6	7498.8	32.0	7579.2	36.3	7715.3	29.5
bankssts	Left	2.53	0.01	2.49	0.01	2.51	0.01	2.53	0.01	2.49	0.01	2.57	0.01	2.58	0.01	2.58	0.01	2.60	0.01	2.58	0.01
bankssts	Right	2.74	0.02	2.71	0.01	2.74	0.02	2.73	0.02	2.70	0.01	2.57	0.01	2.56	0.01	2.57	0.02	2.60	0.01	2.56	0.01
caudalanteriorcingulate	Left	2.53	0.01	2.48	0.01	2.53	0.02	2.50	0.02	2.47	0.02	2.71	0.03	2.66	0.03	2.67	0.03	2.64	0.02	2.57	0.03
caudalanteriorcingulate	Right	2.44	0.02	2.45	0.02	2.44	0.01	2.42	0.01	2.34	0.02	2.63	0.02	2.57	0.02	2.57	0.01	2.57	0.03	2.48	0.02
caudalmiddlefrontal	Left	2.57	0.01	2.54	0.01	2.54	0.01	2.51	0.01	2.50	0.01	2.73	0.01	2.75	0.01	2.75	0.01	2.72	0.01	2.66	0.02
caudalmiddlefrontal	Right	2.59	0.01	2.53	0.01	2.53	0.01	2.50	0.01	2.49	0.01	2.67	0.01	2.64	0.01	2.63	0.01	2.59	0.01	2.54	0.01
cuneus	Left	1.81	0.01	1.81	0.01	1.84	0.02	1.84	0.01	1.84	0.01	2.03	0.02	2.03	0.01	2.00	0.01	1.99	0.01	1.96	0.01
cuneus	Right	1.97	0.01	1.96	0.01	1.97	0.01	1.97	0.01	1.99	0.01	2.00	0.01	2.00	0.00	1.99	0.01	1.99	0.01	1.97	0.01
entorhinal	Left	3.26	0.08	3.18	0.03	3.17	0.08	3.04	0.05	2.95	0.05	3.76	0.05	3.60	0.06	3.49	0.07	3.35	0.05	3.16	0.06
entorhinal	Right	3.42	0.07	3.33	0.03	3.22	0.02	3.11	0.03	3.01	0.04	3.83	0.09	3.61	0.06	3.41	0.07	3.41	0.04	3.36	0.08
frontalpole	Left	2.49	0.01	2.47	0.02	2.49	0.02	2.49	0.01	2.50	0.02	2.80	0.08	2.73	0.05	2.61	0.05	2.62	0.04	2.65	0.03
frontalpole	Right	2.64	0.02	2.63	0.01	2.67	0.02	2.63	0.03	2.68	0.02	2.74	0.02	2.70	0.05	2.73	0.04	2.63	0.04	2.69	0.02
fusiform	Left	2.62	0.01	2.60	0.01	2.59	0.01	2.58	0.01	2.51	0.02	2.90	0.01	2.88	0.01	2.84	0.01	2.79	0.01	2.75	0.02
fusiform	Right	2.73	0.02	2.70	0.01	2.67	0.01	2.65	0.01	2.57	0.01	2.77	0.01	2.76	0.01	2.74	0.02	2.71	0.01	2.65	0.01
inferiorparietal	Left	2.50	0.01	2.46	0.01	2.47	0.01	2.48	0.01	2.46	0.00	2.46	0.01	2.48	0.01	2.43	0.01	2.45	0.01	2.43	0.02
inferiorparietal	Right	2.51	0.01	2.48	0.01	2.45	0.01	2.46	0.02	2.43	0.01	2.58	0.01	2.52	0.01	2.52	0.01	2.51	0.01	2.48	0.01
inferiortemporal	Left	2.76	0.03	2.75	0.01	2.78	0.02	2.76	0.01	2.69	0.01	2.81	0.02	2.81	0.02	2.79	0.01	2.76	0.01	2.77	0.02
inferiortemporal	Right	2.70	0.01	2.65	0.01	2.62	0.01	2.61	0.01	2.59	0.02	2.77	0.01	2.67	0.01	2.64	0.01	2.64	0.01	2.61	0.01
insula	left	2.95	0.03	2.92	0.03	2.89	0.04	2.96	0.02	2.90	0.03	3.01	0.03	3.08	0.03	3.08	0.02	3.03	0.03	2.97	0.03
insula	right	3.10	0.03	3.10	0.02	3.07	0.02	3.07	0.03	3.01	0.03	3.07	0.03	3.14	0.01	3.10	0.02	3.06	0.03	2.95	0.02
isthmuscingulate	Left	2.41	0.02	2.41	0.02	2.41	0.02	2.43	0.01	2.44	0.02	2.30	0.01	2.28	0.02	2.27	0.01	2.26	0.02	2.26	0.01
isthmuscingulate	Right	2.30	0.02	2.28	0.02	2.29	0.02	2.35	0.03	2.32	0.03	2.35	0.03	2.34	0.02	2.32	0.02	2.32	0.02	2.28	0.03
lateraloccipital	Left	2.19	0.01	2.17	0.01	2.18	0.01	2.18	0.01	2.17	0.01	2.36	0.00	2.34	0.01	2.32	0.01	2.35	0.01	2.33	0.01
lateraloccipital	Right	2.33	0.01	2.28	0.01	2.28	0.01	2.28	0.01	2.24	0.01	2.41	0.00	2.35	0.01	2.36	0.01	2.37	0.01	2.34	0.01
lateralorbitofrontal	Left	2.52	0.02	2.47	0.01	2.50	0.02	2.46	0.01	2.34	0.01	2.62	0.01	2.56	0.02	2.55	0.01	2.51	0.01	2.43	0.02
lateralorbitofrontal	Right	2.56	0.02	2.55	0.02	2.54	0.02	2.49	0.01	2.43	0.02	2.67	0.02	2.62	0.01	2.60	0.02	2.56	0.02	2.63	0.02
lingual	Left	1.89	0.01	1.91	0.01	1.92	0.01	1.89	0.01	1.90	0.01	2.10	0.01	2.10	0.01	2.09	0.01	2.08	0.01	2.05	0.01
lingual	Right	1.86	0.02	1.84	0.01	1.86	0.01	1.87	0.01	1.84	0.01	2.07	0.00	2.10	0.01	2.09	0.01	2.07	0.01	2.05	0.01
medialorbitofrontal	Left	2.47	0.01	2.39	0.01	2.42	0.02	2.41	0.01	2.37	0.01	2.52	0.02	2.51	0.02	2.42	0.01	2.39	0.04	2.34	0.03
medialorbitofrontal	Right	2.42	0.01	2.41	0.01	2.41	0.01	2.39	0.01	2.34	0.01	2.62	0.01	2.53	0.03	2.51	0.03	2.47	0.03	2.45	0.03
middletemporal	Left	3.00	0.01	2.96	0.01	2.98	0.01	2.97	0.01	2.94	0.02	2.75	0.01	2.71	0.01	2.70	0.01	2.67	0.01	2.64	0.02
middletemporal	Right	2.90	0.01	2.87	0.01	2.82	0.01	2.84	0.01	2.80	0.01	2.99	0.01	2.89	0.01	2.88	0.01	2.85	0.01	2.82	0.01
paracentral	Left	2.31	0.02	2.31	0.01	2.29	0.01	2.30	0.01	2.28	0.01	2.50	0.01	2.49	0.01	2.46	0.01	2.45	0.01	2.37	0.01
paracentral	Right	2.54	0.01	2.54	0.01	2.51	0.01	2.49	0.01	2.44	0.01	2.61	0.01	2.57	0.01	2.53	0.01	2.48	0.01	2.51	0.01
parahippocampal	Left	2.31	0.02	2.24	0.01	2.23	0.01	2.22	0.02	2.18	0.01	2.81	0.02	2.88	0.02	2.84	0.02	2.79	0.03	2.80	0.02
parahippocampal	Right	2.49	0.02	2.47	0.01	2.46	0.01	2.48	0.01	2.43	0.01	2.83	0.01	2.79	0.02	2.76	0.01	2.74	0.01	2.64	0.02
parsopercularis	Left	2.70	0.01	2.66	0.01	2.67	0.01	2.62	0.01	2.66	0.01	2.59	0.01	2.63	0.01	2.60	0.01	2.51	0.02	2.53	0.01
parsopercularis	Right	2.67	0.01	2.64	0.01	2.63	0.01	2.60	0.01	2.59	0.01	2.60	0.01	2.60	0.01	2.62	0.01	2.55	0.01	2.55	0.01
parsorbitalis	Left	2.57	0.02	2.52	0.01	2.54	0.02	2.52	0.01	2.54	0.02	2.94	0.03	2.99	0.02	2.97	0.02	2.96	0.03	2.92	0.03
parsorbitalis	Right	2.45	0.01	2.47	0.01	2.48	0.02	2.43	0.02	2.44	0.02	2.88	0.02	2.82	0.02	2.79	0.03	2.77	0.01	2.78	0.02
parstriangularis	Left	2.63	0.01	2.61	0.01	2.63	0.01	2.60	0.01	2.60	0.02	2.66	0.01	2.68	0.01	2.63	0.02	2.60	0.02	2.59	0.02
parstriangularis	Right	2.53	0.01	2.53	0.01	2.53	0.01	2.51	0.01	2.47	0.01	2.59	0.02	2.54	0.02	2.57	0.01	2.56	0.01	2.56	0.02
pericalcarine	Left	1.50	0.02	1.59	0.01	1.62	0.01	1.60	0.02	1.62	0.01	1.56	0.00	1.56	0.01	1.55	0.01	1.54	0.01	1.52	0.01
pericalcarine	Right	1.56	0.02	1.56	0.02	1.58	0.01	1.55	0.01	1.58	0.01	1.68	0.01	1.71	0.01	1.71	0.01	1.67	0.01	1.67	0.01
postcentral	Left	2.27	0.01	2.24	0.01	2.25	0.00	2.24	0.01	2.23	0.01	2.13	0.01	2.12	0.01	2.11	0.01	2.09	0.01	2.10	0.01
postcentral	Right	2.18	0.01	2.15	0.01	2.17	0.00	2.15	0.01	2.14	0.01	2.19	0.01	2.15	0.00	2.14	0.01	2.13	0.01	2.13	0.01
posteriorcingulate	Left	2.41	0.02	2.38	0.01	2.37	0.01	2.39	0.01	2.36	0.02	2.46	0.03	2.47	0.01	2.44	0.01	2.40	0.02	2.40	0.02
posteriorcingulate	Right	2.40	0.03	2.42	0.02	2.42	0.01	2.42	0.02	2.40	0.02	2.39	0.01	2.36	0.01	2.34	0.02	2.33	0.02	2.34	0.01
precentral	Left	2.74	0.01	2.70	0.01	2.69	0.01	2.68	0.01	2.66	0.01	2.70	0.01	2.69	0.00	2.66	0.01	2.64	0.00	2.60	0.01
precentral	Right	2.72	0.00	2.68	0.01	2.69	0.01	2.64	0.00	2.62	0.01	2.72	0.01	2.71	0.01	2.70	0.01	2.67	0.00	2.63	0.01
precuneus	Left	2.42	0.00	2.39	0.01	2.39	0.01	2.39	0.01	2.34	0.01	2.42	0.01	2.41	0.01	2.37	0.01	2.36	0.01	2.29	0.00
precuneus	Right	2.40	0.01	2.39	0.01	2.39	0.01	2.36	0.01	2.32	0.01	2.55	0.01	2.52	0.01	2.51	0.01	2.49	0.01	2.45	0.01
rostralanteriorcingulate	Left	2.83	0.03	2.79	0.02	2.79	0.02	2.78	0.01	2.72	0.02	2.80	0.02	2.70	0.02	2.72	0.02	2.70	0.02	2.67	0.03
rostralanteriorcingulate	Right	2.87	0.03	2.88	0.02	2.91	0.02	2.91	0.02	2.80	0.02	3.19	0.08	3.16	0.03	3.08	0.03	3.15	0.04	3.01	0.03
rostralmiddlefrontal	Left	2.42	0.01	2.38	0.01	2.40	0.01	2.40	0.01	2.37	0.01	2.48	0.01	2.47	0.01	2.45	0.01	2.46	0.02	2.41	0.02
rostralmiddlefrontal	Right	2.22	0.01	2.22	0.01	2.22	0.01	2.21	0.01	2.18	0.00	2.34	0.02	2.31	0.01	2.35	0.02	2.30	0.01	2.31	0.01
superiorfrontal	Left	2.73	0.01	2.68	0.01	2.70	0.01	2.69	0.01	2.66	0.01	2.83	0.00	2.85	0.00	2.83	0.01	2.81	0.01	2.75	0.02
superiorfrontal	Right	2.68	0.00	2.66	0.01	2.66	0.01	2.62	0.01	2.61	0.01	2.81	0.01	2.80	0.01	2.81	0.01	2.77	0.01	2.72	0.01
superiorparietal	Left	2.34	0.01	2.33	0.01	2.34	0.01	2.34	0.01	2.32	0.01	2.34	0.00	2.33	0.01	2.30	0.01	2.27	0.01	2.27	0.01
superiorparietal	Right	2.24	0.01	2.22	0.01	2.22	0.00	2.21	0.01	2.17	0.01	2.33	0.01	2.29	0.01	2.28	0.01	2.26	0.01	2.22	0.01
superiortemporal	Left	2.85	0.01	2.80	0.01	2.82	0.01	2.80	0.00	2.76	0.01	2.86	0.01	2.86	0.01	2.85	0.01	2.83	0.01	2.81	0.01
superiortemporal	Right	2.82	0.01	2.81	0.01	2.80	0.00	2.80	0.01	2.77	0.01	3.08	0.00	3.03	0.01	3.04	0.01	3.00	0.01	2.97	0.01
supramarginal	Left	2.58	0.01	2.55	0.01	2.55	0.00	2.55	0.01	2.53	0.01	2.73	0.02	2.73	0.01	2.71	0.01	2.71	0.01	2.68	0.01
supramarginal	Right	2.58	0.01	2.55	0.00	2.53	0.01	2.54	0.02	2.51	0.01	2.68	0.01	2.64	0.01	2.63	0.01	2.61	0.01	2.57	0.01
temporalpole	Left	3.23	0.15	3.49	0.02	3.43	0.04	3.41	0.02	3.30	0.04	3.80	0.10	3.83	0.02	3.82	0.02	3.66	0.03	3.68	0.05
temporalpole	Right	3.41	0.13	3.60	0.03	3.53	0.02	3.47	0.03	3.48	0.03	3.38	0.15	3.45	0.03	3.40	0.04	3.42	0.08	3.40	0.05
transversetemporal	Left	2.36	0.03	2.31	0.01	2.31	0.02	2.24	0.02	2.29	0.01	2.13	0.05	2.16	0.01	2.15	0.01	2.12	0.03	2.10	0.02
transversetemporal	Right	2.55	0.02	2.59	0.01	2.55	0.01	2.54	0.01	2.46	0.02	2.35	0.04	2.37	0.03	2.32	0.02	2.25	0.02	2.32	0.02

Supplementary Table 3

		Subject01										Subject02									
	Protocol	MPR		CSx2		CSx2		CSx8		CSx8		MPR		CSx2		CSx2		CSx8		CSx8	
	Regularization	NA		0.0001 (LR)		0.0012 (HR)		0.0001 (LR)		0.0012 (HR)		NA		0.0001 (LR)		0.0012 (HR)		0.0001 (LR)		0.0012 (HR)	
		MEAN	Std Error	MEAN	Std Error	MEAN	Std Error	MEAN	Std Error	MEAN	Std Error	MEAN	Std Error	MEAN	Std Error	MEAN	Std Error	MEAN	Std Error	MEAN	Std Error
amygdala	Left	1733.7	23.6	1674.2	36.7	1691.6	37.4	1586.9	34.7	1618.8	22.9	1701.7	18.9	1665.1	19.2	1642.8	24.8	1664.7	15.2	1709.9	14.8
amygdala	Right	2000.4	78.4	2101.4	19.3	2076.9	19.1	2097.5	28.6	2166.0	20.8	1653.6	30.7	1766.5	19.6	1734.3	21.8	1720.0	16.5	1781.6	17.2
caudate	Left	3713.1	21.2	3704.4	13.3	3608.0	21.3	3729.2	7.3	3548.8	21.7	3099.5	71.4	3109.3	28.6	3044.6	32.6	3266.0	26.9	3041.5	27.6
caudate	Right	3876.5	18.5	3853.3	18.9	3811.9	13.5	3935.9	28.2	3721.6	12.2	3561.0	25.7	3571.8	38.1	3529.3	16.6	3619.2	22.3	3435.8	29.3
hippocampus	Left	4034.5	24.7	4040.9	25.0	4072.8	16.9	4035.7	25.8	4049.3	7.9	4142.8	36.4	4059.7	22.2	4115.1	33.7	4205.5	21.5	4216.2	25.6
hippocampus	Right	4064.7	51.5	4034.9	22.7	4068.2	31.0	4109.3	35.2	4067.5	23.0	4377.3	45.8	4353.7	23.7	4406.0	13.9	4408.8	34.5	4450.5	21.4
pallidum	Left	1979.4	15.9	1962.6	33.0	1952.3	17.9	1889.6	16.8	1895.2	12.8	1987.0	18.8	1981.0	23.5	1957.8	21.3	1851.7	18.3	1951.8	25.0
pallidum	Right	2046.3	13.4	1999.7	25.1	2036.7	10.2	1836.5	28.1	1882.0	36.7	1889.5	22.8	1916.1	17.9	1910.6	14.0	1783.4	23.5	1920.0	15.1
putamen	Left	4810.1	35.0	4871.8	27.0	4825.8	19.7	4997.3	36.1	4607.2	21.1	4546.7	14.3	4576.8	21.5	4524.3	10.3	4679.9	26.4	4401.6	20.1
putamen	Right	4791.7	24.6	4775.6	30.2	4764.4	20.2	4851.9	33.1	4464.9	44.6	4708.5	6.7	4712.4	30.0	4678.1	18.3	4796.3	26.2	4602.4	19.6
thalamus	Left	8173.7	182.6	8134.4	119.2	8021.8	87.2	8198.9	89.7	8098.9	115.0	7520.5	201.1	7506.6	88.9	7467.8	100.9	8185.2	49.5	7512.3	79.9
thalamus	Right	7441.4	48.4	7488.0	32.3	7348.4	25.2	7967.1	96.9	7501.9	69.7	7484.7	84.8	7509.1	61.0	7436.7	50.1	7867.9	87.3	7377.9	43.8
bankssts	Left	2.53	0.01	2.49	0.01	2.56	0.01	2.45	0.02	2.55	0.01	2.57	0.01	2.58	0.01	2.60	0.01	2.54	0.01	2.61	0.01
bankssts	Right	2.74	0.02	2.73	0.01	2.78	0.01	2.67	0.01	2.74	0.01	2.57	0.01	2.57	0.02	2.61	0.02	2.55	0.02	2.64	0.02
caudalanteriorcingulate	Left	2.53	0.01	2.50	0.01	2.56	0.01	2.42	0.01	2.58	0.01	2.71	0.03	2.65	0.03	2.73	0.03	2.54	0.02	2.76	0.03
caudalanteriorcingulate	Right	2.44	0.02	2.45	0.01	2.48	0.02	2.32	0.01	2.42	0.02	2.63	0.02	2.57	0.01	2.61	0.02	2.41	0.02	2.58	0.01
caudalmiddlefrontal	Left	2.57	0.01	2.53	0.00	2.57	0.01	2.47	0.01	2.48	0.01	2.73	0.01	2.74	0.01	2.78	0.01	2.61	0.01	2.77	0.01
caudalmiddlefrontal	Right	2.59	0.01	2.54	0.00	2.56	0.01	2.45	0.02	2.48	0.01	2.67	0.01	2.62	0.01	2.67	0.01	2.49	0.02	2.61	0.01
cuneus	Left	1.81	0.01	1.82	0.01	1.80	0.00	1.82	0.01	1.79	0.01	2.03	0.02	2.03	0.01	2.03	0.01	1.96	0.01	1.95	0.01
cuneus	Right	1.97	0.01	1.97	0.01	1.97	0.01	1.94	0.02	1.95	0.01	2.00	0.01	2.01	0.01	2.02	0.01	1.96	0.01	1.97	0.01
entorhinal	Left	3.26	0.08	3.20	0.07	3.25	0.05	2.84	0.05	2.99	0.05	3.76	0.05	3.68	0.05	3.69	0.08	3.24	0.08	3.31	0.05
entorhinal	Right	3.42	0.07	3.32	0.05	3.33	0.05	2.88	0.04	3.15	0.03	3.83	0.09	3.55	0.08	3.74	0.04	3.38	0.05	3.55	0.03
frontalpole	Left	2.49	0.01	2.50	0.02	2.53	0.02	2.48	0.03	2.47	0.02	2.80	0.08	2.64	0.03	2.75	0.03	2.51	0.03	2.74	0.03
frontalpole	Right	2.64	0.02	2.62	0.01	2.67	0.02	2.72	0.03	2.69	0.02	2.74	0.02	2.69	0.04	2.73	0.05	2.62	0.03	2.74	0.03
fusiform	Left	2.62	0.01	2.59	0.01	2.66	0.01	2.45	0.01	2.60	0.01	2.90	0.01	2.87	0.01	2.92	0.01	2.67	0.01	2.90	0.02
fusiform	Right	2.73	0.02	2.69	0.01	2.75	0.01	2.52	0.02	2.68	0.01	2.77	0.01	2.77	0.01	2.81	0.01	2.57	0.01	2.79	0.01
inferiorparietal	Left	2.50	0.01	2.45	0.01	2.53	0.01	2.42	0.01	2.54	0.01	2.46	0.01	2.47	0.01	2.50	0.01	2.40	0.01	2.49	0.01
inferiorparietal	Right	2.51	0.01	2.46	0.01	2.52	0.01	2.41	0.01	2.46	0.01	2.58	0.01	2.53	0.01	2.61	0.01	2.44	0.02	2.60	0.02
inferiortemporal	Left	2.76	0.03	2.75	0.01	2.85	0.01	2.59	0.01	2.84	0.01	2.81	0.02	2.82	0.01	2.84	0.02	2.69	0.02	2.88	0.01
inferiortemporal	Right	2.70	0.01	2.64	0.01	2.73	0.01	2.53	0.01	2.69	0.01	2.77	0.01	2.67	0.01	2.73	0.01	2.56	0.01	2.75	0.01
insula	left	2.95	0.03	2.95	0.04	2.95	0.04	2.83	0.01	2.93	0.02	3.01	0.03	3.05	0.03	3.12	0.02	2.95	0.02	3.12	0.04
insula	right	3.10	0.03	3.11	0.02	3.14	0.03	2.87	0.02	3.11	0.03	3.07	0.03	3.08	0.02	3.19	0.02	2.87	0.02	3.16	0.02
isthmuscingulate	Left	2.41	0.02	2.40	0.02	2.46	0.01	2.40	0.03	2.58	0.02	2.30	0.01	2.26	0.01	2.29	0.01	2.22	0.02	2.32	0.02
isthmuscingulate	Right	2.30	0.02	2.29	0.02	2.35	0.02	2.29	0.02	2.49	0.04	2.35	0.03	2.34	0.02	2.40	0.02	2.25	0.01	2.52	0.02
lateraloccipital	Left	2.19	0.01	2.16	0.01	2.20	0.00	2.12	0.01	2.18	0.01	2.36	0.00	2.34	0.01	2.38	0.01	2.29	0.01	2.37	0.01
lateraloccipital	Right	2.33	0.01	2.28	0.00	2.31	0.01	2.20	0.01	2.27	0.01	2.41	0.00	2.36	0.01	2.42	0.01	2.30	0.01	2.39	0.01
lateralorbitofrontal	Left	2.52	0.02	2.47	0.01	2.50	0.01	2.31	0.01	2.41	0.01	2.62	0.01	2.56	0.03	2.59	0.01	2.41	0.01	2.49	0.01
lateralorbitofrontal	Right	2.56	0.02	2.55	0.02	2.61	0.02	2.39	0.02	2.50	0.00	2.67	0.02	2.63	0.01	2.66	0.02	2.61	0.03	2.65	0.01
lingual	Left	1.89	0.01	1.91	0.01	1.90	0.01	1.88	0.01	1.89	0.01	2.10	0.01	2.11	0.00	2.12	0.01	2.03	0.01	2.08	0.01
lingual	Right	1.86	0.02	1.86	0.01	1.86	0.01	1.82	0.01	1.82	0.01	2.07	0.00	2.09	0.01	2.12	0.01	2.04	0.02	2.10	0.01
medialorbitofrontal	Left	2.47	0.01	2.40	0.01	2.42	0.02	2.33	0.01	2.44	0.01	2.52	0.02	2.50	0.02	2.53	0.02	2.30	0.02	2.39	0.02
medialorbitofrontal	Right	2.42	0.01	2.42	0.01	2.45	0.01	2.35	0.02	2.32	0.01	2.62	0.01	2.51	0.02	2.57	0.02	2.42	0.03	2.50	0.01
middletemporal	Left	3.00	0.01	2.94	0.01	3.03	0.01	2.84	0.02	3.01	0.01	2.75	0.01	2.72	0.01	2.72	0.01	2.59	0.01	2.72	0.01
middletemporal	Right	2.90	0.01	2.86	0.01	2.92	0.01	2.76	0.01	2.86	0.01	2.99	0.01	2.90	0.02	2.97	0.01	2.74	0.02	2.93	0.02
paracentral	Left	2.31	0.02	2.31	0.01	2.31	0.01	2.25	0.01	2.21	0.01	2.50	0.01	2.49	0.01	2.49	0.01	2.33	0.01	2.38	0.01
paracentral	Right	2.54	0.01	2.55	0.01	2.55	0.01	2.39	0.01	2.41	0.01	2.61	0.01	2.55	0.01	2.58	0.01	2.45	0.01	2.52	0.01
parahippocampal	Left	2.31	0.02	2.25	0.02	2.27	0.02	2.17	0.02	2.19	0.01	2.81	0.02	2.86	0.02	2.91	0.01	2.74	0.02	2.94	0.01
parahippocampal	Right	2.49	0.02	2.47	0.01	2.47	0.01	2.39	0.01	2.45	0.01	2.83	0.01	2.79	0.02	2.85	0.01	2.55	0.02	2.81	0.01
parsopercularis	Left	2.70	0.01	2.64	0.00	2.73	0.01	2.60	0.02	2.71	0.01	2.59	0.01	2.61	0.01	2.64	0.01	2.48	0.01	2.63	0.02
parsopercularis	Right	2.67	0.01	2.62	0.00	2.65	0.01	2.53	0.02	2.59	0.01	2.60	0.01	2.62	0.01	2.63	0.01	2.47	0.02	2.62	0.01
parsorbitalis	Left	2.57	0.02	2.51	0.01	2.57	0.01	2.50	0.02	2.55	0.01	2.94	0.03	2.97	0.03	3.05	0.02	2.89	0.03	3.03	0.02
parsorbitalis	Right	2.45	0.01	2.47	0.01	2.53	0.02	2.38	0.03	2.48	0.01	2.88	0.02	2.81	0.02	2.91	0.03	2.67	0.02	2.90	0.03
parstriangularis	Left	2.63	0.01	2.61	0.01	2.67	0.01	2.55	0.01	2.62	0.01	2.66	0.01	2.66	0.02	2.70	0.01	2.55	0.02	2.67	0.02
parstriangularis	Right	2.53	0.01	2.51	0.01	2.56	0.01	2.41	0.02	2.52	0.01	2.59	0.02	2.58	0.02	2.62	0.02	2.49	0.02	2.62	0.01
pericalcarine	Left	1.50	0.02	1.60	0.01	1.57	0.01	1.59	0.01	1.58	0.01	1.56	0.00	1.55	0.01	1.54	0.01	1.54	0.01	1.50	0.01
pericalcarine	Right	1.56	0.02	1.54	0.01	1.54	0.01	1.56	0.01	1.56	0.00	1.68	0.01	1.70	0.01	1.69	0.01	1.69	0.01	1.63	0.01
postcentral	Left	2.27	0.01	2.24	0.00	2.27	0.00	2.19	0.01	2.21	0.00	2.13	0.01	2.11	0.01	2.13	0.01	2.09	0.01	2.12	0.01
postcentral	Right	2.18	0.01	2.15	0.01	2.16	0.01	2.13	0.01	2.12	0.00	2.19	0.01	2.15	0.00	2.17	0.01	2.11	0.01	2.12	0.01
posteriorcingulate	Left	2.41	0.02	2.38	0.02	2.42	0.01	2.30	0.01	2.45	0.02	2.46	0.03	2.47	0.03	2.52	0.02	2.35	0.02	2.54	0.01
posteriorcingulate	Right	2.40	0.03	2.43	0.01	2.48	0.02	2.33	0.01	2.48	0.02	2.39	0.01	2.37	0.01	2.39	0.01	2.29	0.01	2.48	0.01
precentral	Left	2.74	0.01	2.69	0.01	2.74	0.01	2.61	0.01	2.65	0.01	2.70	0.01	2.68	0.01	2.70	0.01	2.55	0.01	2.65	0.01
precentral	Right	2.72	0.00	2.68	0.00	2.71	0.01	2.58	0.01	2.61	0.01	2.72	0.01	2.71	0.01	2.74	0.01	2.58	0.01	2.67	0.01
precuneus	Left	2.42	0.00	2.40	0.01	2.42	0.01	2.31	0.01	2.38	0.01	2.42	0.01	2.41	0.01	2.41	0.01	2.26	0.01	2.35	0.01
precuneus	Right	2.40	0.01	2.39	0.01	2.42	0.01	2.28	0.01	2.35	0.01	2.55	0.01	2.51	0.01	2.56	0.01	2.43	0.01	2.52	0.01
rostralanteriorcingulate	Left	2.83	0.03	2.78	0.02	2.81	0.02	2.66	0.01	2.78	0.02	2.80	0.02	2.71	0.02	2.74	0.03	2.58	0.02	2.77	0.02
rostralanteriorcingulate	Right	2.87	0.03	2.87	0.01	2.89	0.02	2.75	0.02	2.95	0.03	3.19	0.08	3.15	0.02	3.21	0.04	2.92	0.04	3.23	0.03
rostralmiddlefrontal	Left	2.42	0.01	2.38	0.01	2.43	0.01	2.35	0.01	2.40	0.01	2.48	0.01	2.46	0.02	2.49	0.01	2.38	0.01	2.48	0.02
rostralmiddlefrontal	Right	2.22	0.01	2.22	0.00	2.22	0.01	2.16	0.01	2.18	0.01	2.34	0.02	2.33	0.02	2.38	0.02	2.25	0.01	2.39	0.02
superiorfrontal	Left	2.73	0.01	2.69	0.01	2.73	0.01	2.60	0.01	2.67	0.01	2.83	0.00	2.84	0.01	2.86	0.00	2.67	0.01	2.85	0.01
superiorfrontal	Right	2.68	0.00	2.65	0.01	2.69	0.01	2.55	0.01	2.64	0.01	2.81	0.01	2.80	0.01	2.86	0.01	2.63	0.01	2.86	0.02
superiorparietal	Left	2.34	0.01	2.33	0.01	2.36	0.01	2.29	0.01	2.31	0.01	2.34	0.00	2.31	0.01	2.35	0.01	2.26	0.01	2.33	0.01
superiorparietal	Right	2.24	0.01	2.22	0.00	2.25	0.01	2.16	0.01	2.17	0.01	2.33	0.01	2.30	0.01	2.31	0.01	2.20	0.01	2.24	0.01
superiortemporal	Left	2.85	0.01	2.81	0.01	2.84	0.01	2.70	0.01	2.79	0.01	2.86	0.01	2.87	0.01	2.90	0.01	2.75	0.01	2.92	0.01
superiortemporal	Right	2.82	0.01	2.81	0.01	2.84	0.01	2.74	0.01	2.80	0.01	3.08	0.00	3.04	0.01	3.08	0.01	2.90	0.01	3.06	0.01
supramarginal	Left	2.58	0.01	2.56	0.01	2.58	0.01	2.46	0.01	2.54	0.01	2.73	0.02	2.71	0.01	2.76	0.01	2.69	0.01	2.75	0.01
supramarginal	Right	2.58	0.01	2.55	0.00	2.59	0.01	2.49	0.01	2.55	0.01	2.68	0.01	2.64	0.00	2.66	0.01	2.53	0.01	2.60	0.01
temporalpole	Left	3.23	0.15	3.47	0.02	3.49	0.05	3.15	0.09	3.47	0.04	3.80	0.10	3.85	0.03	3.83	0.03	3.52	0.04	3.61	0.07
temporalpole	Right	3.41	0.13	3.57	0.03	3.64	0.02	3.45	0.02	3.59	0.02	3.38	0.15	3.48	0.03	3.56	0.03	3.30	0.04	3.52	0.04
transversetemporal	Left	2.36	0.03	2.32	0.02	2.30	0.01	2.24	0.01	2.19	0.02	2.13	0.05	2.16	0.02	2.16	0.01	2.06	0.02	2.08	0.01
transversetemporal	Right	2.55	0.02	2.59	0.01	2.58	0.01	2.40	0.02	2.45	0.01	2.35	0.04	2.40	0.02	2.35	0.02	2.28	0.02	2.29	0.02

Supplementary Table 4
